# Cyclometalated Benzimidazole Osmium(II) Complexes with Antiproliferative Activity in Cancer Cells Disrupt Calcium Homeostasis

**DOI:** 10.1021/acs.inorgchem.3c00501

**Published:** 2023-04-11

**Authors:** Alba Hernández-García, Lenka Marková, María Dolores Santana, Jitka Prachařová, Delia Bautista, Hana Kostrhunová, Vojtěch Novohradský, Viktor Brabec, José Ruiz, Jana Kašpárková

**Affiliations:** † Departamento de Química Inorgánica, Universidad de Murcia, and Murcia BioHealth Research Institute (IMIB-Arrixaca), E-30071 Murcia, Spain; ‡ Czech Academy of Sciences, Institute of Biophysics, CZ-61200 Brno, Czech Republic; § Department of Biophysics, Faculty of Science, Palacky University in Olomouc, CZ-78371 Olomouc, Czech Republic; ∥ SUIC-ACTI, Universidad de Murcia, E-30071 Murcia, Spain

## Abstract

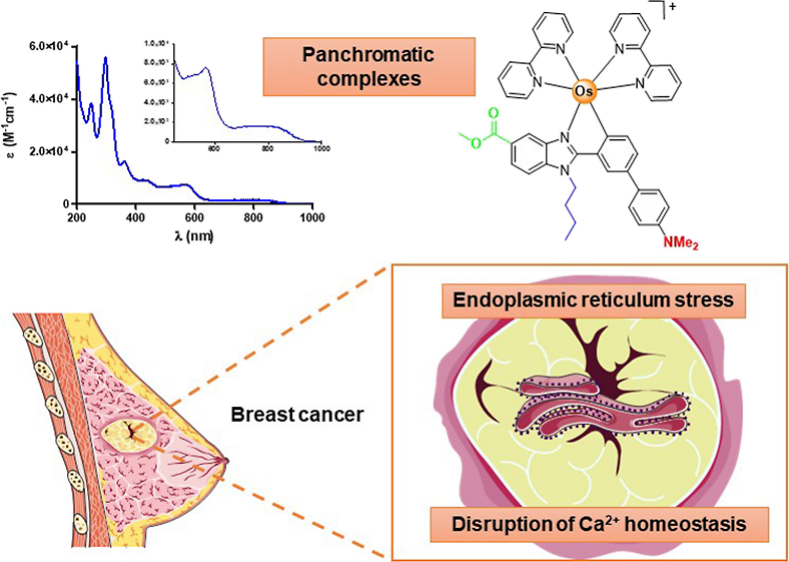

We present the synthesis and characterization of six new heteroleptic osmium(II) complexes of the type [Os(C^N)(N^N)_2_]OTf (N^N = 2,2′-bipyridine and dipyrido[3,2-*d*:2′,3′-*f*]quinoxaline; C^N = deprotonated methyl 1-butyl-2aryl-benzimidazolecarboxylate) with varying substituents in the R3 position of the phenyl ring of the cyclometalating C^N ligand. The new compounds are highly kinetically inert and absorb a full-wavelength range of visible light. An investigation of the antiproliferative activity of the new compounds has been performed using a panel of human cancer and noncancerous 2D cell monolayer cultures under dark conditions and green light irradiation. The results demonstrate that the new Os(II) complexes are markedly more potent than conventional cisplatin. The promising antiproliferative activity of selected Os(II) complexes was also confirmed using 3D multicellular tumor spheroids, which have the characteristics of solid tumors and can mimic the tumor tissue microenvironment. The mechanism of antiproliferative action of complexes has also been investigated and revealed that the investigated Os(II) complexes activate the endoplasmic reticulum stress pathway in cancer cells and disrupt calcium homeostasis.

## Abstract 2

New panchromatic osmium(II) complexes showing high photostability and exhibiting markedly higher antiproliferative activity in the 2D monolayer cancer cell lines and 3D multicellular tumor spheroids than conventional cisplatin; investigated osmium(II) complexes activating the ER stress pathway in cancer cells and disrupting calcium homeostasis; largely unexplored potential of osmium-based complexes for cancer chemotherapy; and hints about the mechanism and targets of systemic osmium toxicity.

## Introduction

**Scheme 1:**
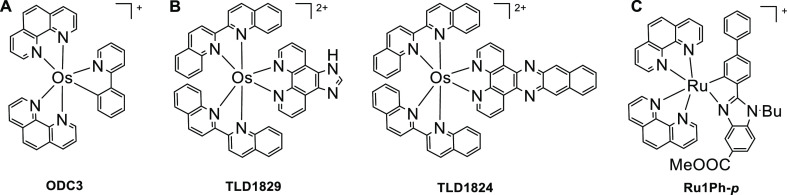
Examples of (A) Os(II) Polypyridyl Complex as an ER Stress Inducer,^14^ (B) Os(II) Panchromatic PSs for PDT,^16^ and (C) Ru(II) PSs for PDT Closely Related to This Work^17^

Cancer is the second leading cause of death worldwide, accounting for nearly 10 million deaths in 2020.^1^ The discovery of the therapeutic properties of cisplatin by Rosenberg had an enormous impact on cancer chemotherapy.^2^ Since then, complexes based not only on platinum but also on other metals have been widely developed, for instance, ruthenium or iridium complexes.^3,4^ Osmium is a member of the platinum group of metals, but its complexes have been far less explored for cancer treatment than those based on platinum or ruthenium. The well-known toxicity of OsO_4_ was partially the cause of the rejection of the use of osmium complexes as anticancer agents.^5^ However, given the clinical success of ruthenium complexes, which entered and progressed into clinical trials, research efforts have been directed toward investigating the therapeutic properties of osmium complexes.^6,7^ First, osmium-based compounds were synthesized as analogues of prototypal ruthenium(III) complexes (NAMI-A, RAPTA-C, and RM-175), showing better properties when changing the metal.^8−10^ Subsequently, the anticancer activity of half-sandwich complexes was explored. Sadler and co-workers developed osmium(II) complexes containing azopyridine ligands, which were active against various cancer cell lines.^11^ Other complexes have shown high levels of cytotoxicity, similar to cisplatin or carboplatin, and their mechanism of action is dependent on the type of structures and oxidation states.^12^ Thus, Os(IV) complexes containing a nitride terminal ligand induce endoplasmic reticulum (ER) stress,^13^ whereas the osmium(II) complex **ODC3** (Scheme 1A) can induce ER stress effectors.^14^ In addition, some half-sandwich osmium(II) complexes able to potentiate the anticancer efficacy of metformin via glucose metabolism reprogramming have been recently reported by Liang et al.^15^

On the other hand, the use of osmium polypyridyl complexes as cellular imaging and photosensitizers (PSs) in photodynamic therapy (PDT) has gained increasing attention more recently. Generally, Os(II) polypyridyl complexes show emission quantum yield and lifetimes lower than other transition-metal complexes, such as ruthenium, iridium, or platinum, for their use in cellular imaging. However, they present some advantages over complexes based on other metals: (1) they present absorption bands over 700 nm, which is within the biological optical window for deep tissue penetration^14,18^ because of the reduction of the energy gap between the ground and excited states, (2) unlike ruthenium-based compounds, osmium d–d states are not accessible by thermal crossover from its excited triplet state,^19^ and (3) they show excellent photostability. In this way, McFarland and co-workers developed the Os PSs **TLD1829** and **TLD1824** (Scheme 1B) containing the auxiliary π-extended 2,2′-biquinoline ligand, which are active by red and NIR light irradiation both under normoxic and hypoxic conditions.^16^ More recently, an osmium–peroxo complex for photoactive therapy of hypoxic tumors has been reported by Zhang et al.^20^ The Zhang group has also investigated a benzimidazole-containing Os(II) complex as a NIR emissive lysosomal tracker,^21^ while the Thomas group has looked at DNA targeting polypyridyl Os(II) complexes as high-resolution contrast probes for TEM.^21^

**Scheme 2:**
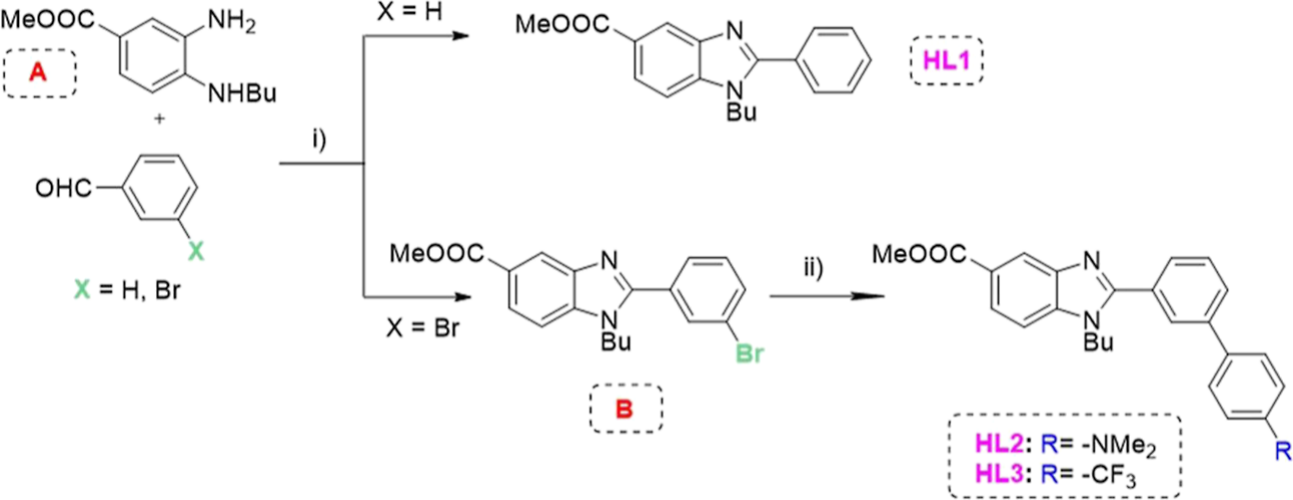
Synthesis of Proligands **HL1**–**HL3**: (i) NaHSO_3_ in Water at 80 °C for 1 h, Followed by Addition of Diamine **A** in EtOH and Heated Overnight at 90 °C; (ii) Corresponding Phenylboronic Acid, Pd(PPh_3_)_4_, and K_2_CO_3_ in Toluene/H_2_O 2:1 and Stirred in a Microwave at 120 °C for 1 h

**Scheme 3:**
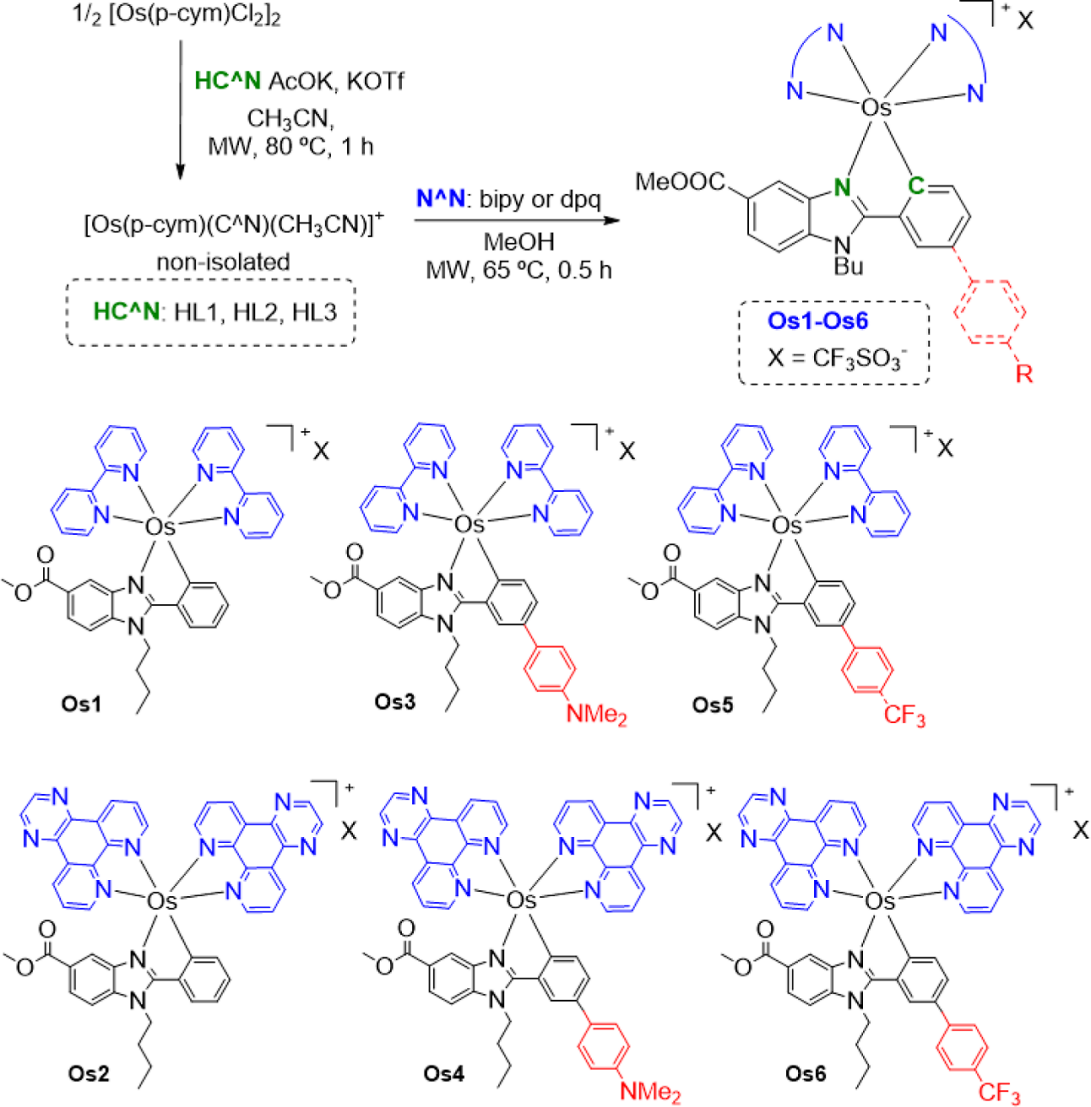
Synthesis of Osmium Complexes **Os1**–**Os6** Investigated in This Work

Herein, we present the development of six new cyclometalated Os(II) complexes **Os1**–**Os6** of the type [Os(C^N)(N^N)_2_]OTf (Scheme 3) to evaluate their therapeutic potential for cancer treatment. The C^N ligand (Scheme 2) is based on a benzimidazole backbone, which is widely used as pharmacophore^22,23^ and has exhibited good (photo)biological results in ruthenium analogues as **Ru1Ph-*p*** (Scheme 1C).^17^ Importantly, the benzimidazole incorporates a masked carboxylic acid function to facilitate further functionalization. We also introduced some substitutions on the aryl group of the C^N ligand to modulate their electronic and biological properties. 2,2′-Bipyridine (bpy) and dipyrido[3,2-*d*:2′,3′-*f*]quinoxaline (dpq) were chosen as N^N ancillary ligands because their implications for bioinorganic chemistry and light-activated metal complexes.^24^ We also report that the investigated Os(II) complexes exhibit a better antiproliferative activity in several human cancer cells than clinically used cisplatin and that they can activate in cancer cells the ER stress pathway and disrupt Ca^2+^ homeostasis.

## Results and Discussion

### Synthesis and Characterization of Proligands and Osmium(II) Complexes

The key intermediate diamine **A** was efficiently obtained from 4-chloro-3-nitrobenzoic acid using reported procedures (Scheme S1).^25^ The preparation of **HL1** and 2-(3-bromophenyl)-benzimidazole derivative **B** (Scheme 2) was achieved by condensing intermediate diamine **A** with benzaldehyde or 3-bromobenzaldehyde, respectively, in ethanol, using sodium bisulfite. The synthesized bromo benzimidazole derivative **B** and the corresponding commercial boronic acid reacted via a Suzuki reaction (Scheme 1) to yield the new pro-ligands **HL2** and **HL3** with a 50–60% yield; NMR spectra showed that the ligands were pure enough for further reactions (Figures S2–S5). The polypyridyl N^N ligand dpq was synthesized following previously reported procedures (Scheme S2).^26,27^

Dark-violet air-stable solid osmium complexes **Os1**–**Os6** (Scheme 3) were obtained via a two-step synthesis following an optimized procedure with respect to the previously reported ruthenium analogues^17^ by reducing the time reaction from 3 days to 1.5 h. First, the cyclometalation reaction was carried out between the dimeric precursor [{Os(η^6^-*p*-cymene)Cl(μ-Cl)}_2_] and the corresponding pro-ligand HC^N, in the presence of an excess of potassium triflate and potassium acetate, in acetonitrile at 80 °C for 1 h. Then, the final Os(II) complex in 20–25% yield was obtained by the reaction of the corresponding non-isolated cyclometalated intermediate [Os(η^6^-*p*-cymene)(C^N)(CH_3_CN)]^+^^28^ and the corresponding N^N ligand (bpy or dpq) in methanol at 65 °C for 30 min (Scheme 3; see the Experimental Section for further details).

All complexes were purified by alumina column chromatography DCM/CH_3_CN 1:1 and fully characterized by ^1^H and ^13^C or DEPT NMR spectroscopy and mass spectrometry (Figures S6–S17). The positive-ion ESI-MS spectra displayed the [M – CF_3_SO_3_]^+^ peaks (Table S1) with the expected isotopic distribution pattern. The ^1^H NMR spectra in CD_3_CN show the presence of many resonances with some overlapping features within the range of 6.0–9.5 ppm for the aromatic hydrogens and the aliphatic peaks from the benzimidazole-based ligands. Complexes were shown to be at least 96% pure by elemental analysis of C, H, N, and S. It is noteworthy that the ^1^H NMR spectra of cyclometalated complexes **Os3**–**Os6** (at 1 mM) showed (Figures S18–S20) significant differences at different compositions of mixtures of DMSO-*d*_6_ and D_2_O. Thus, an upfield shift in some aromatic resonances of the ligands is observed. In addition, an increase in the water content up to 50–70% gives broad signals that lead to the virtual disappearance of the resonances in their ^1^H NMR spectra, with no precipitation being observed in the conditions used (see Figures S19 and S20 for **Os4**–**Os6**), suggesting their self-assembly into supramolecular aggregates. A similar observation was previously found for **Ru1Ph-*p*** (Scheme 1C) and other related cycloruthenated complexes.^17,29^

**Figure 1:**
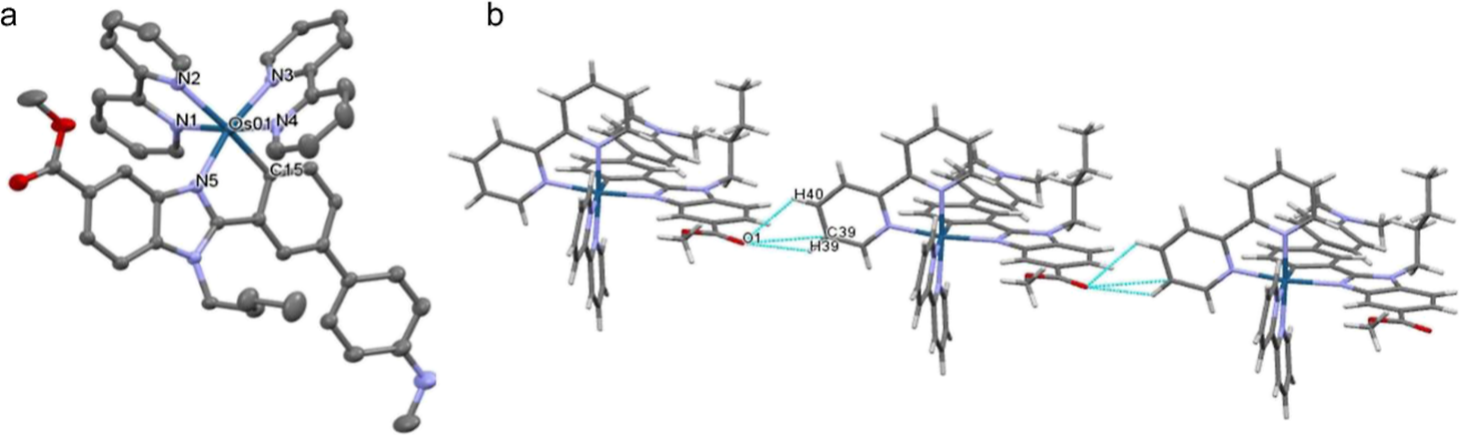
X-ray crystal structure of **Os3** (a) and C–H···O intermolecular interactions (b). Ellipsoids are drawn in (a) at the 50% probability level. Hydrogen atoms, counterions, and solvent molecules are omitted for clarity. Selected bond lengths [Å] and angles [°] for **Os3**: Os(01)–N(1) = 2.0495(19), Os(01)–N(2) = 2.1210(18), Os(01)–N(3) = 2.0334(18), Os(01)–N(4) = 2.040(2), Os(01)–N(5) = 2.0727(18), Os(01)–C(15) = 2.048(2), N(1)–Os(01)–N(2) = 77.01(7), N(3)–Os(01)–N(4) = 78.23(7), and C(15)–Os(01)–N(5) = 77.46(8).

Crystals suitable for X-ray crystallography were obtained by slow diffusion of diethyl ether into a solution of complex **Os3** in dichloromethane. Figure 1a shows the crystal structure of the cation of **Os3** elucidated by single-crystal X-ray analysis. Apart from the cation–anion triflate Coulombic interaction, the packing in the structure of **Os3** is organized by C–H···F and C–H···O intermolecular interactions (Table S4 and Figure 1b). Crystallographic data are listed in Table S3. The complex presents an octahedral geometry around the osmium ion with the four nitrogen atoms of the two bpy ligands (N1–N4) and the remaining positions occupied by the C^N ligand. The nitrogen atom of the C^N ligand (N5) is in *trans* position to the nitrogen N3 of one bpy ligand. The carbon atom of the C^N ligand (C15) is *trans* to the nitrogen N2 of the other bpy. The Os–N_bpy_ bond distances (2.0334–2.1210 Å) are within the range reported for osmium complexes.^14^ The bond distance Os–C15 2.048(2) Å lies in the range reported for osmium compounds containing cyclometalated ligands,^14^ and the *trans* influence of the C atom σ-bound to the metal is reflected in longer Os–N2 distance 2.1210(18) Å.

**Figure 2:**
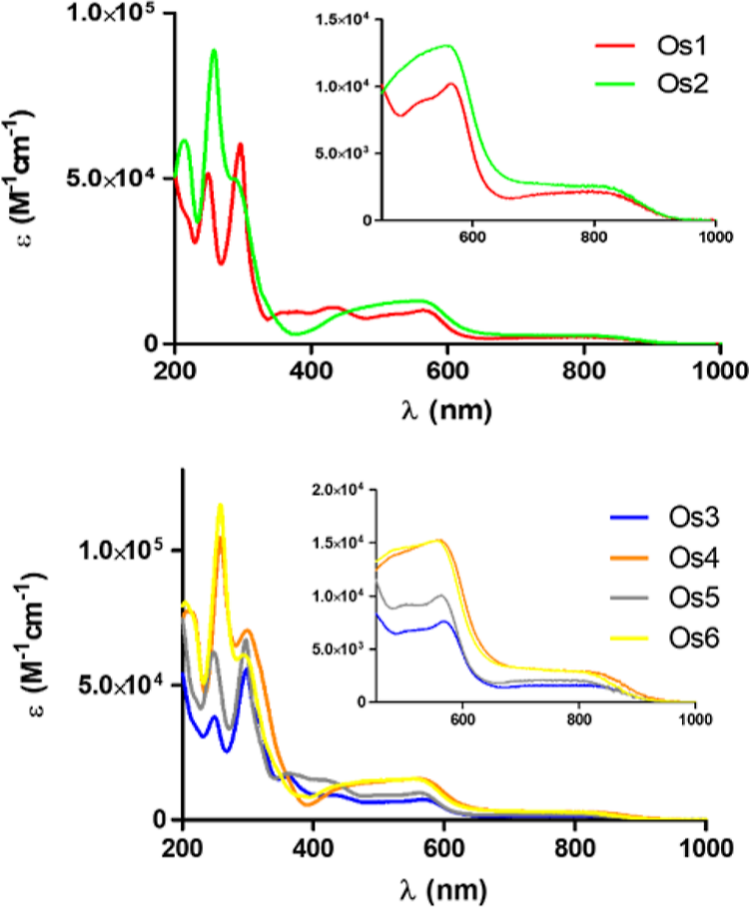
UV–vis absorption spectra of **Os1–Os2** (top) and **Os3–Os6** (bottom) in acetonitrile at room temperature. The inset shows an expansion of the 450–1000 nm region.

The UV–vis absorption spectra of the new osmium complexes were recorded in acetonitrile at room temperature (Figure 2 and Table S2). The new compounds are panchromatic absorbers as they exhibit continuous absorption from 200 to 900 nm, including the NIR-infrared region, which is desirable for PDT. They present strong absorbance between 200 and 400 nm (ε ∼ 120 000–60 000 M^–1^ cm^–1^) due to π–π* electronic transitions of the C^N and N^N ligands. The moderately intense absorption bands in the visible range, between 370 and 630 nm, could be assigned to spin-allowed metal-to-ligand charge transfer (MLCT), whereas the weak and broad spin-forbidden ^3^MLCT absorption bands were seen in the NIR region transitions owing to strong spin–orbit coupling due to the heavy atom effect of osmium (630–900 nm).^21,30^ As observed, subtle structural modifications of the C^N ligand only had a moderate effect on the UV–vis absorption spectra of the corresponding Os complexes.

The absorption spectra of complexes **Os1**, **Os2**, **Os4**, and **Os6** (at 10 μM) in the Roswell Park Memorial Institute medium (RPMI) at 37 °C [a mixture 95:5 (RPMI/DMSO) presented no significant changes over 48 h, as shown in Figure S22], suggesting that they were stable in this growth medium used to grow a variety of mammalian cell types. On the other hand, the UV–vis spectra for **Os3** and **Os5** in the same conditions suggested aggregation and/or slow precipitation, with neither obvious reaction nor isosbestic point being observed. So more diluted conditions (at 1 μM) were used for running their UV–vis spectra (Figure S22).

On the other hand, the new complexes exhibited no photodegradation under white light irradiation (51 mW/cm^2^) during 1.5 h in air-saturated DMSO solution (10 μM), as shown by UV–vis spectroscopy, and their absorption spectra (Figure S23) remain the same before and after light exposure. The capacity of **Os1–Os6** to generate ^1^O_2_ upon irradiation at 520 nm in aerated CH_3_CN was examined by measuring the absorption of 1,3-diphenylisobenzofuran (DPBF) at 413 nm in acetonitrile solution, although extremely low Φ^1^O_2_ (Φ_Δ_) was observed (<0.033; Figures S24–S26, Table S5). Important to note, the analogue ruthenium complex **Ru1Ph-*p*** (Scheme 1C) exhibited much higher quantum yield of singlet oxygen (0.20 in CH_3_CN under green light irradiation)^17^ than the osmium complexes herein reported. Probably the lifetimes of the triplet excited states of these new osmium compounds are shorter because deactivation through nonradiative mechanisms could be operating at higher rates for them.

### Cell Viability

The activity of **Os1**–**Os6** against a panel of human cancer cell lines was assessed by the commonly used 3-(4,5-dimethylthiazol-2-yl)-2,5-diphenyltetrazolium bromide (MTT) assay. The IC_50_ values derived from concentration–response curves are summarized in Table 1 and compared with those found for clinically used cisplatin. All the osmium complexes [Os(C^N)(N^N)_2_]OTf display very low (mostly submicromolar or, rarely, low micromolar) IC_50_ values, which are markedly lower than those yielded by cisplatin. Complex **Os3**, which contains bipyridine as the N^N ligand, was the most potent, whereas complex **Os6**, which contains the dpq as the N^N ligand, was the least active all over the tested cell lines but still markedly more potent than cisplatin. We also conducted viability studies with healthy lung fibroblast MRC5pd30 and normal adult prostatic epithelial cells PNT1A. In general, all osmium complexes were at least as potent toward noncancerous as against cancerous cells.

**Table 1:** IC_50_ Values [μM] of Osmium Complexes **Os1**–**Os6** in a Panel of Human Cell Lines Determined by MTT after 72 h Incubation in the Dark^a^

	**Os1**	**Os2**	**Os3**	**Os4**	**Os5**	**Os6**	cisplatin
PSN1	0.37 ± 0.05	0.45 ± 0.06	0.24 ± 0.03	0.53 ± 0.04	0.57 ± 0.08	1.4 ± 0.1	3.2 ± 0.1
MCF7	0.23 ± 0.03	0.13 ± 0.02	0.15 ± 0.04	0.24 ± 0.01	0.32 ± 0.07	0.92 ± 0.06	14 ± 2
HeLa	0.9 ± 0.1	0.8 ± 0.1	0.6 ± 0.1	0.63 ± 0.06	0.7 ± 0.1	1.1 ± 0.1	14 ± 2
HCT116	0.3 ± 0.1	0.17 ± 0.04	0.12 ± 0.03	0.46 ± 0.08	0.31 ± 0.03	0.71 ± 0.05	9 ± 2
MDA-MB-231	0.26 ± 0.04	0.19 ± 0.03	0.08 ± 0.02	0.28 ± 0.03	0.31± 0.03	0.96 ± 0.04	21 ± 2
OE33	0.31 ± 0.08	0.15 ± 0.03	0.23 ± 0.01	0.45 ± 0.02	0.60 ± 0.06	1.94 ± 0.05	7 ± 1
A375	0.43 ± 0.03	0.27 ± 0.04	0.20 ± 0.02	0.46 ± 0.04	0.55 ± 0.07	1.5 ± 0.2	3.0 ± 0.9
MRC5pd30	0.46 ± 0.05	0.21 ± 0.03	0.19 ± 0.01	0.22 ± 0.02	0.47 ± 0.02	0.7 ± 0.2	12 ± 1
PNT1A	0.19 ± 0.02	0.08 ± 0.01	0.13 ± 0.03	0.40 ± 0.02	0.31 ± 0.01	0.57 ± 0.02	2.4 ± 0.2

a Data represent a mean ± SD from at least three independent experiments, each performed in triplicate.

The data of the MTT test revealed that Os complexes effectively reduce the viability of cells. The MTT assay is based on assaying ongoing cellular (mitochondrial) metabolism, i.e., factors that reflect the number of living cells in the sample. Like that, the MTT assay could not distinguish the effects on cell death (cytotoxicity) on the one hand and cell growth inhibition (cytostatic effect) on the other hand. Therefore, a trypan blue exclusion assay was employed to reveal whether Os complexes inhibit cell proliferation or directly kill the cells. This assay allows cytotoxicity assessment by the direct measurement of the number of dead or damaged cells in a population. The most sensitive MDA-MB-231 cells (Table 1) were treated with **Os3** or **Os6** (the representatives of the family with the highest and the lowest activity), and cells were harvested at certain incubation intervals, stained by trypan blue, and counted. A short incubation time (8 h) was also included to uncover possible acute toxicity.

**Figure 3:**
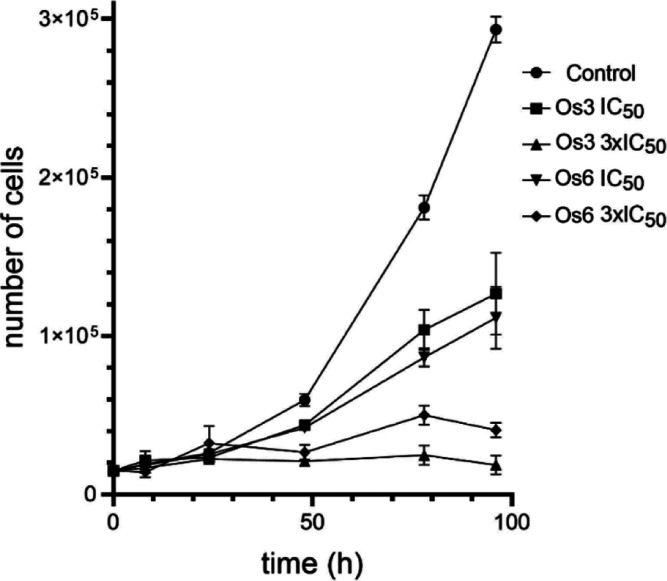
Growth profile of MDA-MB-231 cells untreated or treated with **Os3** or **Os6**. The cells were treated (at *t* = 0) with the equitoxic concentrations of compounds corresponding to IC_50,72h_ or 3 × IC_50,72h_, as determined by MTT (Table 1).

As indicated in Figure 3, the complexes efficiently inhibited cell division and, therefore, the growth of the cell population. However, they show no significant toxic effect in MDA-MB-231 cells (Figure S27). Furthermore, even after a long incubation time (96 h), the number of dead cells was relatively low (ca. 10–20%, Figure S27). Thus, this experiment revealed that Os complexes are not toxic but rather strongly antiproliferative.

**Figure 4:**
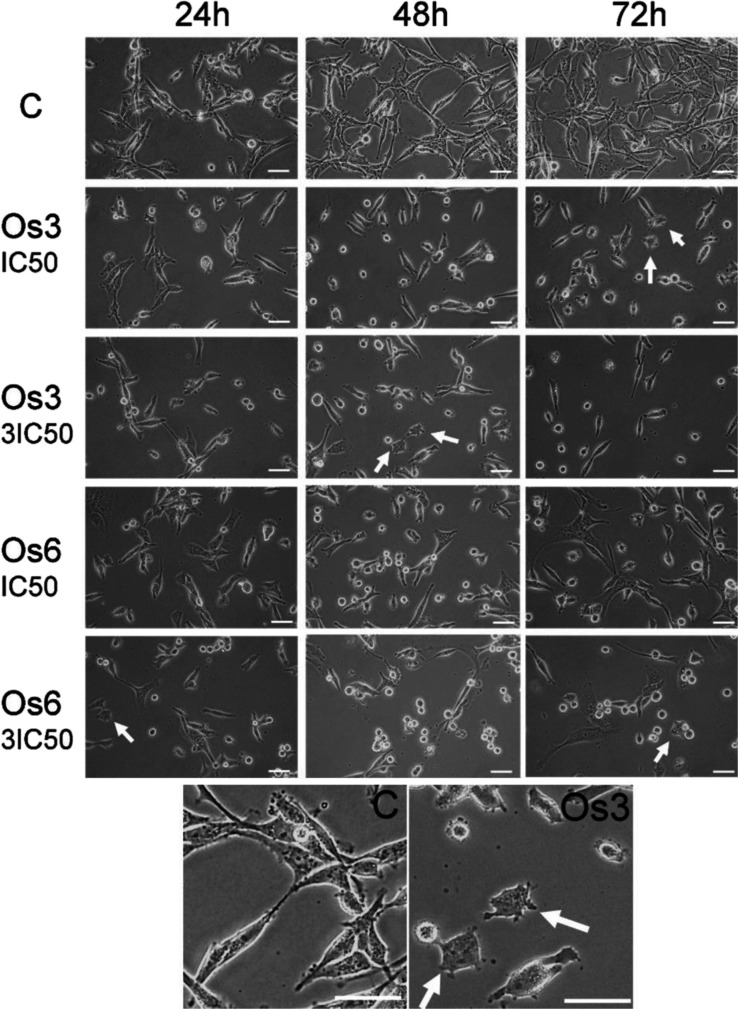
Phase-contrast images of MDA-MB-231 taken at 24, 48, and 72 h of incubation with **Os3** or **Os6** at indicated concentrations. Untreated MDA-MB-231 cells (C) showed an elongated and spindle-shaped morphology with large protruding lamellipodia. After treatment, cells become round-shaped with shorter lamellipodia (the representative cells of this morphology are indicated by arrows). For better resolution, the bottom two panels show a magnified section of the image of untreated cells (C) and cells after treatment with **Os3** (1 × IC_50_), both at 72 h. In all images, the scale bar represents 50 μm.

Although the cells were not dead yet with a compromised cell membrane in the time scale of the experiment, their morphology was significantly affected by the treatment with **Os3** or **Os6**, as indicated in Figure 4. This revealed physiological changes in MDA-MB-231 cells caused by **Os3** and **Os6**, especially at longer times and/or higher concentrations. Thus, not only was cell growth inhibited but the cells also showed significant changes in their morphology. Changes in the cell structure are thought to be due to the rearrangement of cytoskeletal proteins and are critically involved in the transformation of adhesion and polarized cell migration required for the success of the metastatic process.^31−33^

### Antiproliferative Effect of Os Complexes in Three-Dimensional (3D) Cell Culture

**Figure 5:**
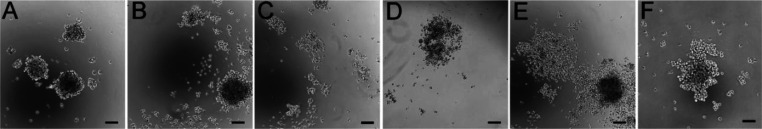
Representative images of MDA-MB-231 spheroids untreated (A) or treated with complex **Os3** at concentration 0.62 (B), 1.25 (C), 2.5 μM (D), complex **Os6** at 2.5 μM (E), and cisplatin at 50 μM (F). The concentrations used in panels (B,E) roughly correspond to the IC_50_ values found for **Os3** and **Os6**, respectively. Scale bars represent 100 μm.

Although cell cultures grown in 2D monolayer are the most frequently used for testing the biological activity of various metallodrugs, three-dimensional (3D) cell cultures are assumed to be a more representative model for in vitro anticancer drug screening.^34,35^ Cells growing in 3D cultures possess several features of “in vivo” tumors, such as hypoxia, cell–cell interaction, drug penetration, and production/deposition of the extracellular matrix.^36,37^ Therefore, the effect of the two selected complexes, **Os3** and **Os6**, was also tested on 3D cultures of MDA-MB-231 cells to provide more relevant data on its antitumor activity (Figures 5 and S28).

MDA-MB-231 mammospheres were markedly more sensitive to the investigated osmium complexes than to conventional cisplatin, and complex **Os3** containing the electron-donating substituent NMe_2_ was significantly more potent compared to **Os6** (Figure 5 and Table 2). These results confirm the trend in antiproliferative activity found for 2D monolayer MDA-MB-231 cells (Table 1); however, the difference between the antiproliferative activities of the two complexes is less pronounced. This may reflect properties typical for 3D but not for 2D cultures, such as different penetration to the cells inside 3D spheroids.

**Table 2:** IC_50_ Values^a^ (μM) Obtained for the Investigated Os Compounds in MDA-MB-231 Derived Mammospheres Determined Using CellTiter-Glo 3D Cell Viability Assay

	IC_50_ (μM)
**Os3**	0.82 ± 0.05
**Os6**	2.2 ± 0.1
cisplatin	59 ± 6

a Data represent mean values ± SD from three independent experiments.

### Determination of Intracellular Reactive Oxygen Species

In general, the redox activity of Os(II) complexes is associated with the formation of reactive oxygen species (ROS) in cells.^12^ In this context, many previously reported osmium compounds had been shown to induce oxidative stress in cancer cells.^38−42^ To assess the causative role of oxidative stress in the biological effects of the complexes tested in this study, the generation of ROS by complexes **Os3** and **Os6** was investigated by the CellROX green assay. Although menadiol (a positive control) was significantly active in this experiment, levels of ROS in MDA-MB-231 cells indicated upon exposure to a wide range of drug concentrations were not elevated for both Os complexes (Figure S29). This suggests that increased ROS production was unlikely to play a role in the biological activity of these Os(II) benzimidazole complexes.

### Effect on Calcium Homeostasis

Based on the abovementioned results, oxidative stress due to ROS formation was ruled out as a possible mechanism of action. Thus, the biological effects must therefore be related to another mechanism. A number of previously reported osmium (and ruthenium) compounds are assumed to induce cell death through activation of the ER stress pathway.^14,43−45^ These findings led us to investigate the possibility that Os(II) benzimidazole complexes may induce ER stress as well.

The ER is a cellular organelle crucial for peptide synthesis and maturation, lipid synthesis, detoxification of chemicals, and, importantly, calcium (Ca^2+^) storage.^46^ An elevated endoplasmic Ca^2+^ level relative to the cytosol is of critical importance for cells because of its role in regulating a variety of cellular processes. Frequently, ER stress leads to the release of Ca^2+^ from the ER lumen;^47^ therefore, the changes in Ca^2+^ levels are considered a reliable marker for ER stress.^48^

**Figure 6:**
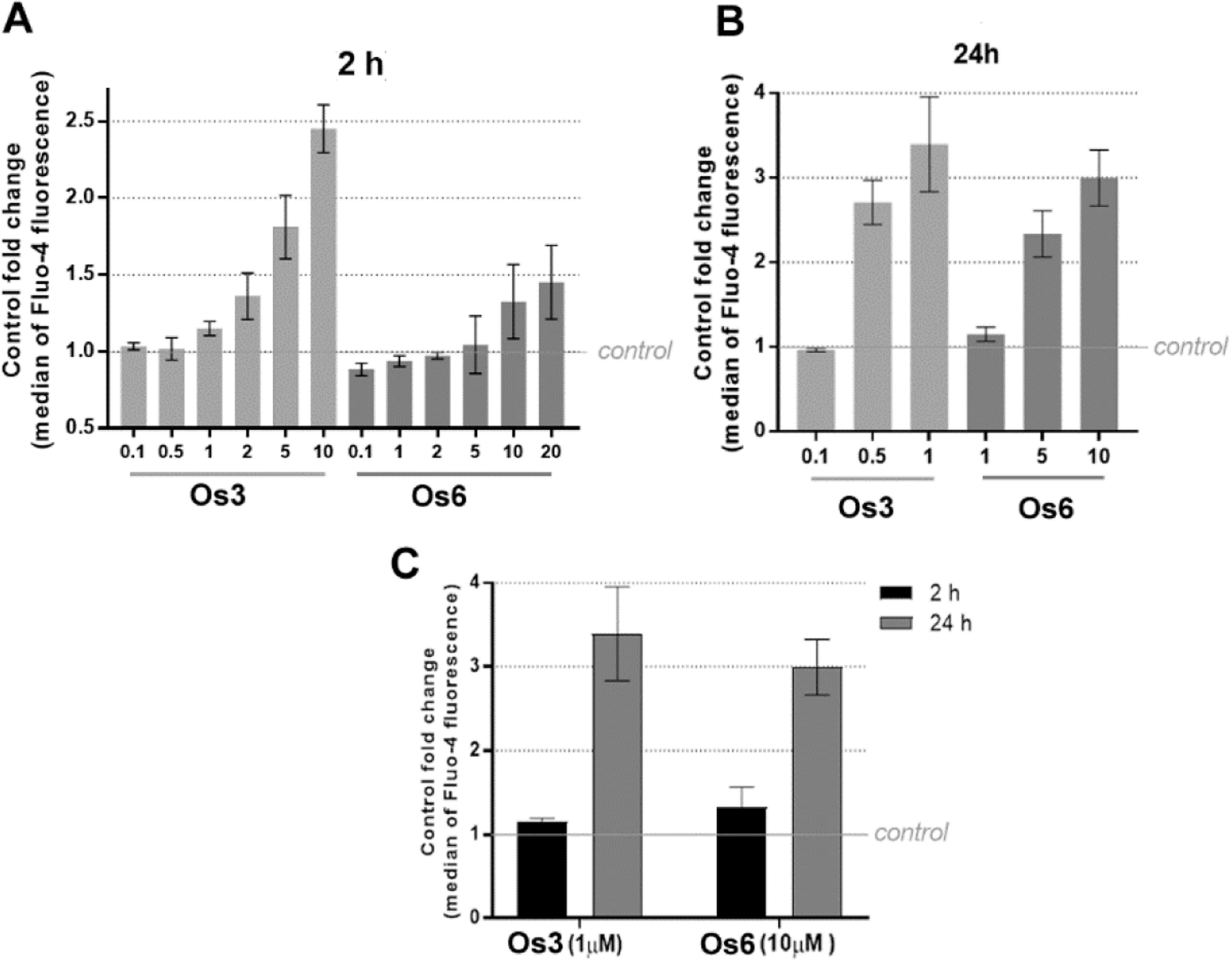
Cytoplasmic calcium level in MDA-MB-231 cells determined by the Fluo-4 reagent. MDA-MB-231 cells were treated with indicated concentrations of Os complexes. Cytoplasmic calcium level was measured immediately after the treatment. (A,B) Cells were treated with various concentrations of **Os3** or **Os6** for 2 (A) or 24 h (B) in the dark. (C) Comparison of the values obtained for equipotent concentrations of **Os3** and **Os6**. The values represent the fluorescence intensity (median) related to the value found for control, untreated cells.

Calcium release from ER into the cytoplasm was studied using the calcium-sensitive fluorescent probe Fluo-4 AM. As indicated in Figure 5, incubation with the investigated Os complexes resulted in a significant elevation of cytoplasmic Ca^2+^ concentration even after a short incubation time; the effect was both concentration (Figure 6A,B) and time (Figure 6C) dependent. Interestingly, **Os3** was roughly 10-fold more effective than **Os6** (Figure 6C), reflecting their antiproliferative potency (IC_50_ values differ by ca. 10-fold, Table 1). This suggests that the antiproliferative effects of the Os complexes tested in this work may be closely related to the loss of Ca^2+^ homeostasis and disruption of Ca^2+^ signaling.

**Figure 7:**
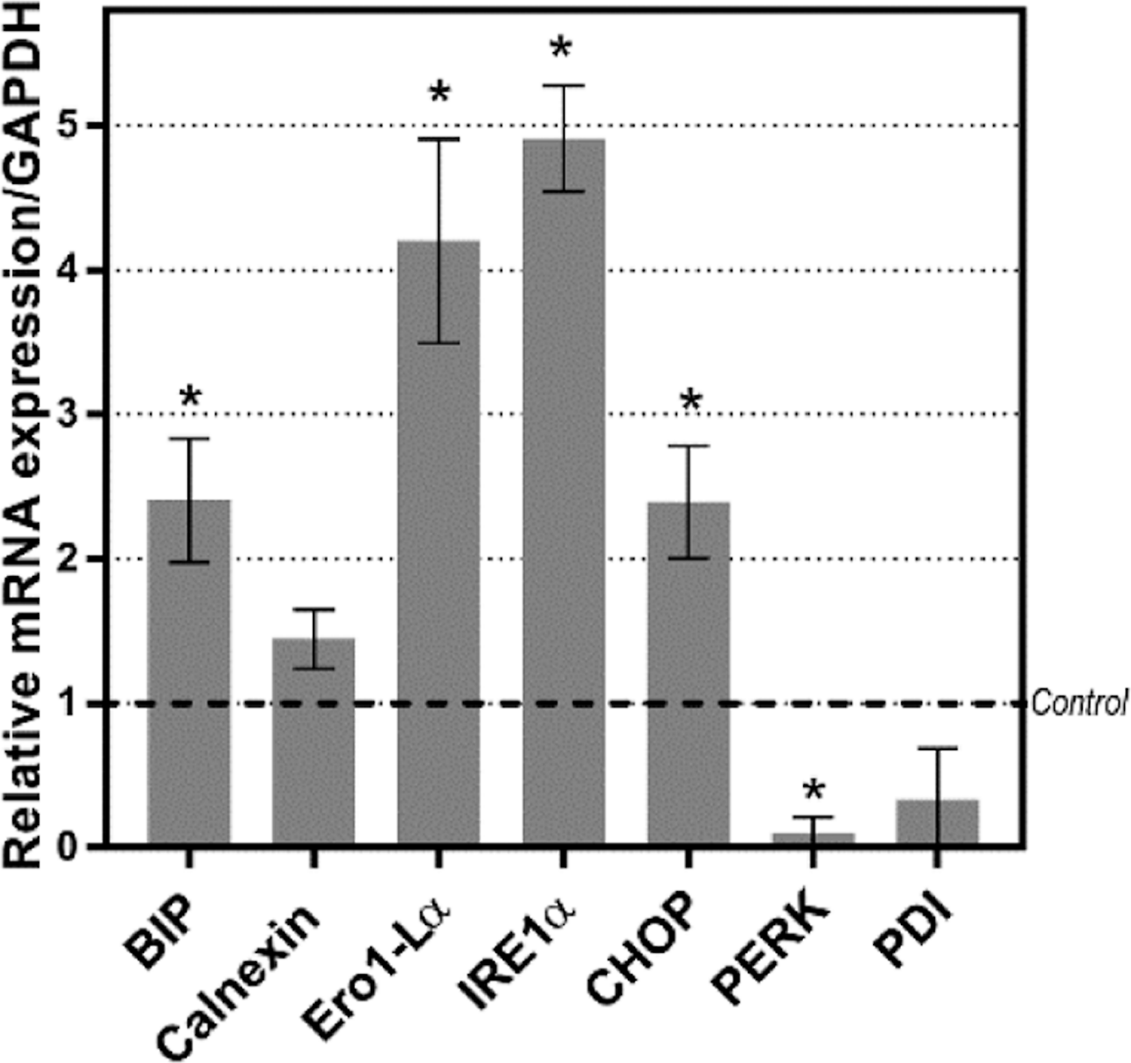
qRT-PCR of the ER stress markers in MDA-MB-231 cells treated with the tested **Os3** complex. Cells were treated for 24 h at the concentration corresponding to 3 × IC_50,72h_. Relative quantification of gene expression was calculated using the 2^–ΔΔ*Ct*^ method. The GAPDH gene was used as the endogenous reference control, and samples isolated from the untreated cells were used as the arbitrary calibrators. Data were subjected to statistical analysis using Student’s *t* test and a significant difference (*p ≤ 0.01) from untreated control samples (dashed line).

However, the treatment-associated elevation of cytoplasmic Ca^2+^ may depend on interference with calcium homeostasis systems other than ER. Therefore, to further support the view that Os(II) benzimidazole belongs to the ER stress inductors, we analyzed the expression of key biomarkers of the ER stress pathway.^13^ Using qRT-PCR, we found that the expression of several biomarkers of this pathway was significantly affected by Os complexes, such as Bip, Ero1-Lα, IRE1α, and CHOP (Figures 7 and S30), so that the level of particular mRNAs was 2.5–4.9 above the level found in untreated cells. The effect was dependent on incubation time and was very similar for both, **Os3** and **Os6** when applied in equitoxic concentrations (Figure S30).

**Figure 8:**
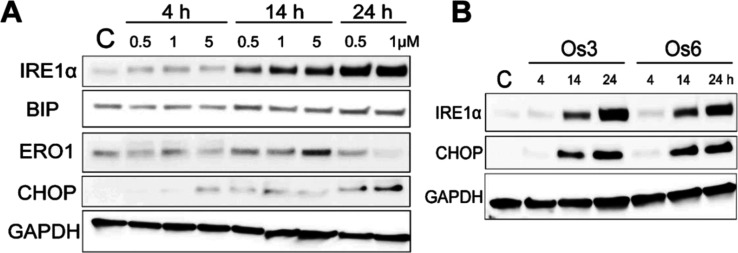
(A) Western blot images of ER stress markers and GAPDH in MDA-MB-231 cells non-treated (C) or treated with **Os3** at 0.5, 1, or 5 μM concentrations for 4, 14, or 24 h. (B) Western blot images of ER stress markers and GAPDH in MDA-MB-231 cells non-treated (C) or treated with **Os3** or **Os6** at their equitoxic concentrations corresponding to 3 × IC_50,72h_ for 4, 14, and 24 h. For quantitative evaluation, see Figure S31.

To confirm that the changes in mRNA levels are translated into the proteins, Western blotting analysis was performed. The results clearly confirm the significantly increased expression of selected ER markers (Figure 8). The effect was both concentration- and time-dependent (Figures 8 and S31). The quantitative analyses of the latter results revealed that particularly Ire1α and CHOP were increased dramatically; the protein levels in cells treated for 24 h with equitoxic concentrations (3 × IC_50_) of **Os 3** or **Os6** were increased ca. 15–20 times (Ire1α) or even 130 times (CHOP), as compared to untreated cells. Hence, the intensity of the induction suggested that the investigated Os complexes are efficient inducers of at least some markers of ER stress, which might be biologically relevant.

### Phototoxicity Testing

The photoactivatibility of the complexes has been tested on three different human cancer cell lines, namely, cervical adenocarcinoma HeLa, esophageal carcinoma OE33, and melanoma A375 cells. These lines were chosen because these types of tumor tissues are readily available for PDT treatment.

The phototoxicity data are summarized in Table 3. As indicated, the antiproliferative activity of all investigated complexes was promoted by irradiation with green light under normoxic conditions. Among the tested compounds, **Os1** was the most sensitive to irradiation, demonstrating the highest phototoxicity index (∼4) in all cell lines. As the complexes in the dark do not show significant selectivity for tumor versus noncancerous cells (Table 1), their photopotentiation may represent a considerable advantage. During photodynamic chemotherapy, only tumor tissue is irradiated selectively so that the photopotentiation at the site of the tumor can significantly increase the difference between the effects on cancer and healthy (nonirradiated) tissue.

**Table 3:** IC_50_ Values^a^ (μM) Obtained for HeLa, OE33, and A375 Cancer Cells Treated with the Investigated Os Complexes in the Dark or after Irradiation by Green Light (1 h, λ_max_ = 533 nm, 77 ± 3 W m^–2^) Determined by the MTT Assay

	HeLa (normoxia)			HeLa (hypoxia)			OE33 (normoxia)			A375 (normoxia)		
	dark	irrad	PI	dark	irrad	PI	dark	irrad	PI	dark	irrad	PI
**Os1**	2.6 ± 0.4	0.6 ± 0.1	4.3	3.0 ± 0.4	1.5± 0.4	2.0	0.9 ± 0.1	0.21 ± 0.06	4.3	0.8 ± 0.1	0.19 ± 0.02	4.2
**Os2**	0.8 ± 0.2	0.31 ± 0.04	2.6	0.7 ± 0.1	0.6± 0.1	1.2	0.50 ± 0.08	0.19 ± 0.03	2.6	0.4 ± 0.1	0.19 ± 0.05	2.1
**Os3**	2.0 ± 0.3	0.9 ± 0.3	2.2	3.6 ± 0.5	2.2 ± 0.2	1.6	1.5 ± 0.4	0.9 ± 0.2	1.7	0.8 ± 0.2	0.44 ± 0.08	1.8
**Os4**	1.0 ± 0.3	0.5 ± 0.1	2.0	0.5 ± 0.1	0.41 ± 0.04	1.2	6 ± 1	3.3 ± 0.3	1.8	0.9 ± 0.2	0.5 ± 0.1	1.8
**Os5**	4.2 ± 0.8	1.6 ± 0.4	2.6	3.4 ± 0.6	2.4 ± 0.4	1.4	4 ± 1	2.0 ± 0.6	2.0	0.8 ± 0.2	0.6 ± 0.1	1.3
**Os6**	2.0 ± 0.6	1.5 ± 0.5	1.3	1.3 ± 0.4	1.1 ± 0.2	1.2	6 ± 1	5 ± 1	1.2	1.4 ± 0.1	1.4 ± 0.3	1.0

a Cells were treated with Os compounds for 1 h in the dark followed by 1 h irradiation (or shame irradiation). The assay was evaluated after 70 h post-incubation in a drug-free medium. Normoxia (21% O_2,_ 5% CO_2_), hypoxia (3% O_2,_ 5% CO_2_), PI (phototoxic index) = [IC_50_]_dark_/[IC_50_]_irrad._ The results are expressed as mean values ± SD from at least three independent experiments, each made in triplicate.

The antiproliferative activity of Os(II) complexes was also determined under hypoxic conditions (3% O_2_) in the model HeLa cells. All investigated Os compounds showed significant activities even in hypoxia (Table 3), both in the dark and after irradiation. Irradiation also led to a photopotentiation in these hypoxic conditions, although the PI was slightly lower for most complexes than that determined under a normal oxygen concentration. In the case of **Os4**, **Os5**, and **Os6**, the antiproliferative activity determined after irradiation under hypoxic conditions was even similar to that determined under normoxic conditions. The results thus suggest that the investigated Os complexes might represent candidate compounds for the therapeutic treatment of hypoxic tumors.

## Conclusions

In this work, we have synthesized and characterized six octahedral robust panchromatic osmium(II) complexes of the type [Os(C^N)(N^N)_2_]OTf that show high photostability. An investigation of the antiproliferative activity of the new compounds in the 2D monolayer cancer cell lines and 3D multicellular tumor spheroids under dark conditions and green light irradiation demonstrated that the new Os(II) complexes, particularly complex **Os3**, are markedly more potent than clinically used cisplatin. The mechanism of antiproliferative action of complexes has also been investigated and revealed that increased ROS production was unlikely to play a role in the biological activity of these Os(II) benzimidazole complexes. On the other hand, under dark conditions, the investigated Os(II) complexes can activate the ER stress pathway in cancer cells and disrupt calcium homeostasis. In addition to the design of new Os(II) compounds of biological importance, further expansion of the theoretical background for a deeper understanding of osmium biology was also achieved. The presence of an ester as a handle for further functionalization on the Os complexes developed herein will optimize their cancer cell selectivity by varying the nature of the targeting unit. It is also of note that some of the new Os(II) complexes were photoactivated under hypoxic conditions (3% in O_2_) in HeLa cells, although only slightly (Table 3). Thus, this observation does not qualify these Os complexes as suitable candidates for clinical photochemotherapy. On the other hand, because of the modular structure of these scaffolds, they could enable the preparation of new libraries of osmium PSs for photocytotoxicity and unlock the possibility of exciting it at up to 900 nm. Overall, this study highlights the largely unexplored potential of osmium-based complexes for cancer chemotherapy and provides hints about the mechanism and targets of systemic osmium toxicity.

## Experimental Section

### General Remarks

Synthesis-grade solvents were employed in all cases. Deuterated solvents acetonitrile-*d*_3_ and chloroform-*d* were purchased from Euriso-top. The diamine precursor **A** was obtained, as previously described.^17^ All other reagents were obtained from commercial sources. All compounds are >96% pure by elemental analysis. Nuclear magnetic resonance (NMR) spectra were recorded at 25 °C on a Bruker AC 300E, Bruker AV 400, or Bruker AV 600 NMR spectrometer. The corresponding deuterated solvent was used as an internal reference. The solvents used were acetonitrile and DMSO, and their internal reference was 1.94 and 2.50 ppm for ^1^H spectra, respectively, and 1.32 and 39.52 ppm for ^13^C. Elemental analyses were performed on a LECO’s CHNS-932 Elemental Analyzer. UV–vis spectroscopy was carried out on a PerkinElmer Lambda 750 S spectrometer with operating software. ESI mass (positive mode) analyses were carried out on an HPLC/MS TOF 6220.

### Synthesis and Characterization of Intermediates, Ligands, and Proligands

#### Modified Procedure for the Synthesis of **HL1** and 2-(3-Bromophenyl)-benzimidazole Derivative **B**

Respective aldehyde (3 mmol) and sodium bisulfite (637 mg, 6 mmol) were dissolved in water and stirred at 80 °C for 1 h. Then, the diamine **A** (220 mg, 3 mmol) was dissolved in EtOH and added to the solution. The mixture was stirred overnight at 90 °C. EtOH was removed under reduced pressure, and an extraction was performed with dichloromethane (3 × 15 mL). The final organic phase was dried with anhydrous magnesium sulfate. The solvent was removed under reduced pressure, and the corresponding product was obtained by adding ethyl acetate and hexane.

**HL1** was obtained as a yellow oil (95%); this modified procedure improves the yield previously published by us.^17^**HL1** was used without further purification to synthesize the corresponding osmium complexes.

**B** was obtained as a white solid (504 mg, 43.6%).

^1^H NMR (401 MHz, chloroform-*d*) δ: 8.54–8.49 (m, 1H), 8.06 (dd, *J* = 8.6, 1.6 Hz, 1H), 7.90 (t, *J* = 1.8 Hz, 1H), 7.66 (dddd, *J* = 8.6, 7.8, 1.8, 1.0 Hz, 2H), 7.47–7.40 (m, 2H), 4.30–4.20 (m, 2H), 3.96 (s, 3H), 1.87–1.74 (m, 2H), 1.29 (h, *J* = 7.3 Hz, 2H), 0.89 (t, *J* = 7.4 Hz, 3H).

#### Synthesis of **HL2**

Intermediate **B** (387 mg, 1 mmol) and 4-(dimethylamino)phenylboronic acid (198 mg, 1.2 mmol) were added to a microwave tube. Tetrakis(triphenylphosphine)palladium(0) (58 mg, 0.05 mmol) was used as a catalyst, and potassium carbonate (414.6, 3 mmol) was used as a base. The mixture was dissolved in 6 mL of toluene/H_2_O 2:1 and stirred under microwave at 120 °C for 1 h. Then, dichloromethane and water were added to perform an extraction (3 × 15 mL of DCM). The organic phase was dried with anhydrous magnesium sulfate, and the solvent was removed under reduced pressure. The crude solid was purified in a silica column with a solvent mixture of 6:4 hexane/ethyl acetate, the solvent was removed, and the **HL2** was precipitated with hexane.

**HL2** was obtained as a white solid (264 mg, 62%).

^1^H NMR (401 MHz, chloroform-*d*): δ 8.55 (dd, *J* = 1.6, 0.6 Hz, 1H), 8.05 (dd, *J* = 8.6, 1.6 Hz, 1H), 7.90 (m, 1H), 7.77–7.68 (m, 1H), 7.61–7.54 (m, 4H), 7.45 (dd, *J* = 8.6, 0.7 Hz, 1H), 6.87–6.77 (m, 2H), 4.29 (t, *J* = 7.66 Hz, 2H), 3.96 (s, 3H), 3.01 (s, 6H), 1.88–1.76 (m, 2H), 1.27 (m, 2H), 0.86 (t, *J* = 7.3 Hz, 3H).

^13^C{^1^H} NMR (101 MHz, CDCl_3_): δ 167.60, 155.39, 149.89, 142.00, 138.48, 129.42, 128.42, 128.01, 127.36, 126.95, 125.18, 124.81, 121.96, 113.37, 110.16, 77.48, 52.33, 45.11, 41.01, 31.95, 20.06, 13.66.

ESI-MS (positive ion mode): *m*/*z* = 428.2339 [M + H]^+^ calcd *m*/*z* 428.2380. Anal. Calcd for C_27_H_29_N_3_O_2_: C, 75.85; H, 6.84; N, 9.83. Found: C, 75.71; H, 6.83; N, 9.72.

#### Synthesis of **HL3**

Intermediate **B** (387, 1 mmol), 4-(trifluoromethyl)phenylboronic acid (227.9 mg, 1.2 mmol), tetrakis(triphenylphosphine)palladium(0) (58 mg, 0.05 mmol), and potassium carbonate (414.6, 3 mmol) were dissolved in 6 mL of toluene/H_2_O 2:1 and stirred under microwave at 120 °C for 1 h. After extraction with dichloromethane (3 × 15 mL), the organic phase was dried with anhydrous magnesium sulfate, and the solvent was removed under reduced pressure. The crude solid was purified in a silica column with solvent mixture hexane: ethyl acetate 8:2 as the eluent. Then, the solvent was removed, and hexane was added to obtain a white solid (240 mg, 53%).

^1^H NMR (300 MHz, chloroform-*d*): δ 8.55 (dd, *J* = 1.6, 0.6 Hz, 1H), 8.07 (dd, *J* = 8.5, 1.6 Hz, 1H), 7.98 (m, 1H), 7.80–7.77 (m, 1H), 7.77–7.73 (m, 4H), 7.72 (t, *J* = 1.6 Hz, 1H), 7.65 (m, 1H), 7.46 (dd, *J* = 8.5, 0.7 Hz, 1H), 4.35–4.25 (m, 2H), 3.97 (s, 3H), 1.95–1.73 (m, 2H), 1.30 (h, *J* = 7.4 Hz, 2H), 0.87 (t, *J* = 7.3 Hz, 3H).

^13^C{^1^H} NMR (75 MHz, CDCl_3_): δ 167.60, 154.96, 143.67, 142.71, 140.55, 138.85, 131.01, 129.45, 128.87, 128.72, 128.40, 127.52, 125.91, 125.86, 124.71, 124.45, 122.41, 109.85, 52.13, 44.84, 31.89, 19.93, 13.51.

ESI-MS (positive ion mode): *m*/*z* = 453.1790 [M + H]^+^ calcd *m*/*z* 453.1780. Anal. Calcd for C_26_H_23_N_2_F_3_O_2_: C, 69.02; H, 5.12; N, 6.19. Found: C, 69.06; H, 5.15; N, 5.98.

### Synthesis and Characterization of Os(II) Complexes

All osmium complexes were prepared following the same synthetic route. [Os(η^6^-*p*-cymene)Cl_2_]_2_ (80 mg, 0.1 mmol), potassium acetate (59 mg, 0.6 mmol), potassium triflate (75.3 mg, 0.4 mmol), and the corresponding proligand (0.2 mmol), previously synthesized, were added to a microwave tube and stirred at 80 °C for 1 h. Then, the mixture was filtered, and the solvent was removed under reduced pressure. The crude product was dissolved in methanol and used without further purification. Then, the corresponding N^N ligand (0.3 mmol) was added to the solution and stirred at 65 °C for 30 min. The solvent was removed, and the crude solid was purified in alumina column chromatography with a gradient from DCM/CH_3_CN 9:1 to DCM/CH_3_CN 1:1. Finally, osmium complexes were recrystallized using dichloromethane and hexane and filtered to obtain a dark-violet solid.

#### [Os(bpy)_2_(**L1**)]CF_3_SO_3_ (**Os1**)

**HL1** (61.67 mg, 0.2 mmol) was used as a proligand, and 2,2′-bipyridine (bpy) (62.5 mg, 0.4 mmol) was used as the N^N ligand.

**Os1**. Dark-violet solid. Isolated yield: 22%. ^1^H NMR (600 MHz, CD_3_CN): δ (ppm) 8.41 (d, *J* = 8.4, 1H), 8.36–8.27 (m, 3H), 7.96 (d, *J* = 7.8 Hz, 1H), 7.93–7.86 (m, 2H), 7.84–7.78 (m, 2H), 7.65 (s, 1H), 7.63–7.55 (m, 2H), 7.48 (t, *J* = 7.8 Hz, 1H), 7.40 (t, *J* = 7.8 Hz, 1H), 7.34 (t, *J* = 6.2 Hz, 1H), 7.08 (t, *J* = 6.2 Hz, 1H), 7.02 (t, *J* = 6.0 Hz, 1H), 6.99 (t, *J* = 6.0 Hz, 1H), 6.81–6.76 (m, 2H), 6.32 (s, 1H), 6.00 (s, 1H), 4.71 (t, *J* = 7.5 Hz, 2H), 3.70 (s, 2H), 2.01–1.91 (m, 2H), 1.41 (m, 2H), 0.93 (t, *J* = 7.4 Hz, 3H). ^13^C{^1^H} NMR (151 MHz, CD_3_CN): δ 167.83, 167.15, 163.00, 161.43, 159.68, 155.31, 151.87, 151.56, 150.62, 141.52, 140.62, 136.61, 134.39, 134.07, 133.85, 129.90, 128.57, 128.44, 128.03, 126.25, 125.88, 125.11, 124.53, 124.40, 124.31, 124.07, 123.26, 121.13, 117.01, 111.56, 52.34, 45.82, 32.40, 20.62, 14.07. ESI-MS (positive ion mode, CH_3_CN): *m*/*z* = 811.2449 [M]^+^ calcd *m*/*z* 811.2431. Anal. Calcd for C_40_H_35_N_6_O_5_F_3_SOs: C, 50.10; H, 3.68; N, 8.76; S, 3.34. Found: C, 50.14; H, 3.86; N, 8.52; S, 3.41 (%).

#### [Os(dpq)_2_(**L1**)]CF_3_SO_3_ (**Os2**)

**HL1** (61.67 mg, 0.2 mmol) and dpq (93 mg, 0.4 mmol) were used following the procedure previously described.

Pure **Os2** was obtained as a dark-violet solid following the same purification procedure.

**Os2**. Dark-violet solid. Isolated yield: 25%. ^1^H NMR (401 MHz, CD_3_CN): δ (ppm) 9.30 (d, *J* = 8.1 Hz, 1H), 9.14–9.09 (m, 3H), 9.08 (d, *J* = 2.2 Hz, 1H), 8.96 (d, *J* = 8.1 Hz, 1H), 8.88 (d, *J* = 8.1 Hz, 1H), 8.80 (d, *J* = 8.1 Hz, 1H), 8.49 (d, *J* = 5.6 Hz, 1H), 8.44 (d, *J* = 5.6 Hz, 1H), 8.33 (d, *J* = 5.3 Hz, 1H), 8.19 (d, *J* = 5.5 Hz, 1H), 7.95 (d, *J* = 7.8 Hz, 1H), 7.69 (m, 2H), 7.56 (dd, *J* = 8.1, 5.4 Hz, 1H), 7.51–7.45 (m, 2H), 7.37 (dd, *J* = 8.1, 5.5 Hz, 1H), 6.88 (t, *J* = 7.3 Hz, 1H), 6.70 (t, *J* = 7.1 Hz, 1H), 6.33–6.30 (m, 2H), 4.72 (t, *J* = 7.5 Hz, 2H), 3.60 (s, 3H), 1.99 (m, 2H), 1.43 (m, 2H), 0.94 (t, *J* = 7.3 Hz, 3H). ^13^C{^1^H} NMR (151 MHz, CD_3_CN): δ 177.15, 167.83, 166.91, 156.87, 155.50, 154.15, 153.79, 153.29, 153.24, 152.46, 152.14, 147.24, 147.16, 147.08, 141.70, 141.43, 141.32, 141.19, 140.98, 140.37, 137.43, 136.39, 132.33, 131.08, 130.92, 130.79, 130.73, 130.68, 130.05, 129.94, 129.54, 128.25, 128.05, 127.95, 127.10, 125.99, 124.87, 122.44, 117.02, 111.70, 52.29, 45.93, 32.43, 20.66, 14.09. ESI-MS (positive ion mode, CH_3_CN): *m*/*z* = 963.2564 [M]^+^ calcd *m*/*z* 963.2554. Anal. Calcd for C_48_H_35_N_10_O_5_F_3_SOs: C, 51.88; H, 3.17; N, 12.61; S, 2.84. Found: C, 50.72; H, 3.07; N, 12.59; S, 2.79 (%).

#### [Os(bpy)_2_(**L2**)]CF_3_SO_3_ (**Os3**)

**HL2** (85 mg, 0.2 mmol) was used as the proligand to obtain the corresponding crude product intermediate, and bipyridine (62.5 mg, 0.4 mmol) was used as the N^N ligand to obtain the final dark-violet complex.

**Os3**. Dark-violet solid. Isolated yield: 22%. ^1^H NMR (401 MHz, CD_3_CN): δ (ppm) 8.44 (m, 2H), 8.35 (m, 2H), 8.19 (s, 1H), 8.01–7.87 (m, 2H), 7.81 (s, 1H), 7.76 (d, *J* = 8.6 Hz, 1H), 7.61 (m, 3H), 7.51 (s, 1H), 7.44 (m, 2H), 7.35 (d, *J* = 8.6 Hz, 3H), 7.13 (m, 2H), 7.06–6.95 (m, 2H), 6.82 (*J* = 8.7 Hz, 2H), 6.21 (s, 1H), 5.59 (s, 1H), 4.78 (t, *J* = 7.6 Hz, 2H), 3.69 (s, 3H), 3.03 (s, 6H), 2.04 (m, 2H), 1.45 (m, 2H), 0.97 (t, *J* = 7.3 Hz, 3H). ESI-MS (positive ion mode, CH_3_CN): *m*/*z* = 930.3078 [M]^+^ calcd *m*/*z* 930.3166. Anal. Calcd for C_48_H_44_N_7_O_5_F_3_SOs: C, 53.47; H, 4.11; N, 9.09; S, 2.97. Found: C, 53.49; H, 4.06; N, 9.13; S, 3.13 (%).

#### [Os(dpq)_2_(**L2**)]CF_3_SO_3_ (**Os4**)

**HL2** (85 mg, 0.2 mmol) was used as a proligand, and dpq (93 mg, 0.4 mmol) was used as the N^N ligand to prepare the final violet complex.

**Os4**. Dark-violet solid. Isolated yield: 19.5%. ^1^H NMR (401 MHz, CD_3_CN): δ (ppm): 9.33 (dd, *J* = 8.2, 1.2 Hz, 1H), 9.17–9.09 (m, 4H), 9.00 (dd, *J* = 8.1, 1.2 Hz, 1H), 8.93 (dd, *J* = 8.1, 1.1 Hz, 1H), 8.88 (dd, *J* = 8.2, 1.2 Hz, 1H), 8.59 (dd, *J* = 5.6, 1.3 Hz, 1H), 8.50 (d, *J* = 5.6 Hz, 1H), 8.35 (dd, *J* = 5.4, 1.3 Hz, 1H), 8.21 (d, *J* = 5.7 Hz, 1H), 8.10 (d, *J* = 1.8 Hz, 1H), 7.77–7.67 (m, 2H), 7.62 (dd, *J* = 8.2, 5.5 Hz, 1H), 7.61–7.49 (m, 2H), 7.46–7.36 (m, 3H), 6.95 (dd, *J* = 7.9, 1.7 Hz, 1H), 6.81–6.73 (m, 2H), 6.36–6.28 (m, 2H), 4.80 (t, *J* = 7.4 Hz, 2H), 3.61 (s, 3H), 2.93 (s, 6H), 2.07 (m, 2H), 1.57–1.40 (m, 2H), 0.98 (t, *J* = 7.4 Hz, 3H). ^13^C{^1^H} NMR (101 MHz, CD_3_CN): δ (ppm): 155.93, 152.34, 149.98, 146.30, 146.20, 146.13, 140.70, 140.52, 140.37, 140.28, 140.05, 139.42, 131.38, 130.12, 129.99, 129.84, 129.73, 129.22, 129.03, 127.27, 127.12, 126.98, 126.82, 125.01, 123.94, 116.03, 112.88, 110.69, 51.28, 45.08, 39.76, 31.37, 19.84, 13.13. ESI-MS (positive ion mode, CH_3_CN): *m*/*z* = 1082.3292 [M]^+^ calcd *m*/*z*: 1082.3289. Anal. Calcd for C_56_H_44_N_11_O_5_F_3_SOs: C, 54.67; H, 3.6; N, 12.52; S, 2.61. Found: C, 54.61; H, 3.62; N, 12.55; S, 2.72 (%).

#### [Os(bpy)_2_(**L3**)]CF_3_SO_3_ (**Os5**)

**HL3** (90 mg, 0.2 mmol) was used as the C^N ligand, and bipyridine (62.5 mg, 0.4 mmol) was used as the N^N ligand. After column purification, **Os5** was recrystallized using dichloromethane and ethyl ether (instead of hexane) and filtered to obtain a dark-violet solid.

**Os5**. Dark-violet solid. Isolated yield: 29%. ^1^H NMR (401 MHz, DMSO-*d*_6_): δ 8.77 (d, *J* = 8.2 Hz, 1H), 8.74–8.60 (m, 3H), 8.25 (s, 1H), 8.05 (t, *J* = 7.8 Hz, 1H), 7.90 (d, *J* = 8.7 Hz, 1H), 7.87–7.69 (m, 8H), 7.64 (t, *J* = 7.7 Hz, 1H), 7.54 (t, *J* = 5.9 Hz, 3H), 7.32 (s, 1H), 7.22 (s, 3H), 6.37–5.86 (m, 2H), 5.02–4.90 (m, 2H), 3.69 (s, 3H), 1.98–1.91 (m, 2H), 1.46–1.26 (m, 2H), 0.88 (t, *J* = 7.4 z, 3H). ESI-MS (positive ion mode, CH_3_CN): *m*/*z* = 955.2641 [M]^+^ calcd *m*/*z* 955.2618. Anal. Calcd for C_47_H_38_F_6_N_6_O_5_SOs: C, 51.17; H, 3.47; N, 7.62; S, 2.91. Found: C, 51.11; H, 3.39; N, 7.36; S, 2.96 (%).

#### [Os(dpq)_2_(**L3**)]CF_3_SO_3_ (**Os6**)

**HL3** (90 mg, 0.2 mmol) was used as a proligand, and dpq was used as the N^N ligand (93 mg, 0.4 mmol). Pure **Os6** was obtained as a dark-violet solid.

**Os6**. Dark-violet solid. Isolated yield: 19.9%. ^1^H NMR (600 MHz, CD_3_CN): δ (ppm): 9.34 (d, *J* = 8.2, 1H), 9.17–9.10 (m, 4H), 9.02 (d, *J* = 8.1 Hz, 1H), 8.97 (d, *J* = 8.2 Hz, 1H), 8.89 (d, *J* = 8.2 Hz, 1H), 8.52 (d, *J* = 5.6 Hz, 1H), 8.46 (m, 1H), 8.34–8.30 (m, 1H), 8.19 (m, 2H), 7.77–7.69 (m, 6H), 7.66–7.60 (m, 1H), 7.59–7.53 (m, 2H), 7.42 (dd, *J* = 8.1, 5.5 Hz, 1H), 7.04 (d, *J* = 7.9 Hz, 1H), 6.36 (s, 1H), 6.32 (s, 1H), 4.82 (t, *J* = 7.6 Hz, 3H), 3.61 (s, 3H), 2.11–2.02 (m, 2H), 1.54–1.41 (m, 2H), 0.96 (t, *J* = 7.5 Hz, 3H). ESI-MS (positive ion mode, CH_3_CN): *m*/*z* = 1107.2747 [M]^+^ calcd *m*/*z*: 1107.2741. Anal. Calcd for C_55_H_38_N_10_F_6_O_5_SOs: C, 52.63; H, 3.05; N, 11.16; S, 2.55. Found: C, 52.53; H, 2.93; N, 11.01; S, 2.62 (%).

### X-ray Structure Determinations

Intensities were registered at low temperatures on a Bruker D8QUEST diffractometer using monochromated Mo Kα radiation (λ = 0.71073 Å). Absorption corrections were based on multi-scans (program SADABS).^49^ Structures were refined anisotropically using SHELXL-2018.^50^ Hydrogen atoms were included using rigid methyl groups or a riding model.

### Special Features

The structure contains poorly resolved regions of residual electron density; this could not be adequately modeled and so was “removed” using the program SQUEEZE, which is part of the PLATON system.^51^ The void volume per cell was 539 Å^3^, with a void electron count per cell of 160. This additional solvent was not taken into account when calculating derived parameters, such as the formula weight, because the nature of the solvent was uncertain.

### Stability in Cell Culture Medium

The stability of complexes in the cell culture medium was evaluated by UV–vis spectra at *t* = 0 and after 48 h at 37 °C. Complexes were dissolved in RPMI (5% DMSO) with 10 μM of concentration for **Os1**, **Os2**, **Os4**, and **Os6** and 1 μM for **Os3** and **Os5**. Moreover, experimental details of the studies of HPLC-MS of all complexes in RPMI are described in the Supporting Information.

### Photostability with White Light Irradiation

The complexes were dissolved in an air-saturated DMSO solution with 10 μM of the concentration. For measuring the photostability, osmium compounds were irradiated with white light (51 mW/cm^2^) for 1.5 h. The UV–vis spectra of complexes were recorded from 250 to 1000 nm.

### Singlet Oxygen Yields

The procedure was adapted from the literature.^52,53^ Osmium complexes were dissolved in air-saturated CH_3_CN solution with an approximate absorbance of 0.06 at 520 nm. The solutions were irradiated with green light, and the UV–visible spectra of DPBF were recorded every 30 s for 4 min and a half. [Ru(bpy)_3_](PF_6_)_2_ was used as a reference, in which absorbance at 520 nm and with 50 μM is 0.052. A PBS solution of DPBF without the PS was used as a negative control.

Singlet oxygen quantum yields were obtained using the equation
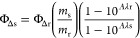
where Φ_Δr_ is the singlet oxygen quantum yield of the reference in acetonitrile (0.57),^31^ Φ_Δs_ is the singlet oxygen quantum yield of each sample, *m* are the slopes of samples, and reference and *A*λ are the absorbance of complexes and reference at irradiation wavelength (520 nm).

### Biological Studies

Stock solutions of the Os complexes were prepared by dissolving the compounds in DMSO to a final concentration of 5 mM and subsequently diluted by culture medium to the required concentration. To avoid DMSO toxicity, the final DMSO concentration in the cell culture medium did not exceed 0.1% (v/v).

### Cell Lines and Culture Conditions

HeLa human cervix adenocarcinoma cells, OE33 human Caucasian oesophageal carcinoma, A375 human skin melanoma cells, highly invasive breast carcinoma MDA-MB-231 cells, human pancreatic cancer cell line PSN1, immortalized normal adult human prostatic cells PNT1A, and human MRC-5 pd30 cells derived from normal lung tissue were purchased from ECACC (UK). Human colorectal carcinoma cells (HCT-116) and human breast cancer (MCF-7) cells were kindly supplied by Professor B. Keppler, University of Vienna (Austria).

PSN1, OE33, and PNT1A cells were cultured in RPMI medium (Biosera), and all the other cells used in this work were kept in DMEM medium (high glucose, 4.5 g L^–1^, Biosera); both media were supplemented with gentamycin (50 μg mL^–1^) and 10% heat-inactivated FBS (Biosera). The cells were cultivated at 37 °C in a humidified 5% CO_2_ atmosphere and periodically subcultured to keep appropriate plating density.

### Antiproliferative Activity

Antiproliferative activities of the investigated complexes in 2D cell cultures were determined using a commonly used MTT assay. Briefly, the cells seeded in 96-well tissue culture plates at a density of 5 × 10^3^ cells/well in 100 μL of medium were incubated overnight at 37 °C in a 5% CO_2_ humidified atmosphere. Afterward, the cells were treated with the tested compounds in the concentration range of 0 to 100 μM and incubated for 72 h. After that, 20 μL of a freshly diluted MTT solution (1.25 mg mL^–1^ in PBS, Calbiochem, Darmstadt, Germany) was added, followed by a 4 h incubation at 37 °C, 5% CO_2_. The medium was then removed, and the resulting formazan product was dissolved in 100 μL of DMSO. The cell viability was evaluated by measurement of the absorbance at 570 nm using an absorbance reader (SPARK TECAN, SCHOELLER). At least three independent experiments were performed, each of them made in triplicate. The antiproliferative effectivities were expressed as IC_50_ values calculated from the curves constructed by plotting relative absorbance (% of untreated control) versus drug concentration (μM) (IC_50_ = concentration of the agent inhibiting cell population growth by 50%). The concentration of Os complexes present in the medium during treatment was verified by flameless atomic absorption spectrometry.

Effects of the Os compounds were also determined in the 3D culture of MDA-MB-231 cells. The cells were seeded in ultra-low attachment 96-well plates at a density of 5000 cells/well in DMEM/F12 Ham medium supplemented with epidermal growth factor (20 ng mL^–1^), BSA (1.5 mg mL^–1^), and B-27 supplement (2%, Invitrogen, Thermo Fisher Scientific Inc., MA, USA) and allowed to grow for 4 days. The resulting 96 h old spheroids were treated with the investigated compounds for an additional 72 h. Treated spheroids were then monitored and transferred to a black plate. One volume of the CellTiter-Glo 3D reagent (Promega, Prague, Czech Republic) was added and incubated for 30 min. This 3D assay reagent measures ATP as an indicator of viability and generates a luminescence signal measured by a Tecan SPARK reader. IC_50_ values were determined from the dose–response curves. To determine IC_50_ values, the CellTiter-Glo 3D cell viability assay (Promega, Prague, Czech Republic) was used according to the manufacturer’s protocol.

### Trypan Blue Exclusion Assay

MDA-MB-231 cells, seeded in 24-well plates in DMEM medium, were treated with Os complexes and incubated at 37 °C in a 5% CO_2_. At certain incubation intervals, cells were harvested, stained by trypan blue, and counted by using BioRad TC10 Automated cell counter.

### Determination of Intracellular ROS

Intracellular ROS were quantified in MDA-MB-231 cells. Cells were seeded on six-well plates at a density of 3 × 10^5^ cells per well. After 2 days, the cells were treated with Os complexes (0.1, 1, 5, or 10 μM) or menadiol (100 μM) for 2 h. After the incubation, the cells were harvested, and 5 μM CellROX green reagent (Life Technologies) was added to the cells and incubated for 30 min at 37 °C. Cells were then washed with PBS, and the fluorescence intensity (exc. 488 nm, emis: 520 nm) was measured by the flow cytometer Cell Stream (Amnis).

### Determination of the Cytoplasmic Ca^2+^ Level

Calcium release from ER into the cytoplasm was studied using the calcium-sensitive fluorescent probe Fluo-4 AM (Invitrogen, Thermo Fisher Scientific). MDA-MB-231 cells were seeded on six-well plates at a density of 3 × 10^5^ cells per well. After 2 days, the cells were treated with Os complexes at their various concentrations or ionomycin (5 μM) for 2 or 24 h. Cells were harvested, stained with the Fluo-4 reagent (5 μM), and incubated for 30 min at 37 °C. Cells were then washed with PBS, and the fluorescence intensity (exc. 488 nm, emis: 520 nm) was measured by flow cytometer Cell Stream (Amnis).

### qRT-PCR

MDA-MB-231 cells were seeded on six-well plates at a density of 3 × 10^5^ cells and incubated overnight. Cells were then treated with Os complexes **Os3** or **Os6** (at various concentrations) for 4, 14, or 24 h. Following washing, the cells were harvested, and total RNA was isolated using NucleoSpin RNA columns (Machery Nagel, GE). One-step qPCR combining reverse transcription followed by amplification thermal cycling was applied using Luna universal one-step RT-qPCR (New England BioLabs, MA, USA). Experiments were performed on Illumina Eco real-time PCR (Illumina, CA, USA). The thermal profile was as follows: reverse transcription for 10 min at 55 °C, initial denaturation for 1 min at 95 °C, followed by 43 thermal cycles of denaturation for 10 s at 95 °C, and extension for 30 s at 60 °C. Primer sequences:

GAPDH-F: GTCTCCTCTGACTTCAACAGCG, GAPDH-R: ACCACCCTGTTGCTGTAGCCAA; BiP(GRP78)-F: CTGTCCAGGCTGGTGTGCTCT, BiP(GRP78)-R: CTTGGTAGGCACCACTGTGTTC, Calnexin-F: GCTGGTTAGATGATGAGCCTGAG, Calnexin-R: ACACCACATCCAGGAGCTGACT, ERO1A-F: GAAGGCTGTTCTTCAGTGGACC, ERO1A-R: CCCTTGTAACCAGTGTAGCGCT, IRE1-F: CCGAACGTGATCCGCTACTTCT, IRE1-R: CGCAAAGTCCTTCTGCTCCACA, DDIT3 (CHOP)–F: GGTATGAGGACCTGCAAGAGGT, DDIT3(CHOP)-R: CTTGTGACCTCTGCTGGTTCTG, PERK-F: GTCCCAAGGCTTTGGAATCTGTC, PERK-R: CCTACCAAGACAGGAGTTCTGG, PDIA2-F: GCTGCTGTTTGTCAACCAGACG, PDIA2-R: CCTCAGCCTTGAGTCCAAAGTAC purified primers were obtained from Generi Biotech (Czech Republic). GAPDH was used as an internal control. The relative expression of mRNA is represented as a fold increase (2^–ΔΔ*Ct*^).

### Immunoblotting Experiments

MDA-MB-231 cells were seeded at a density of 3 × 10^5^ cells/dish, grown overnight, and incubated with Os complexes **Os3** or **Os6** (at various concentrations) for 4, 14, or 24 h. Following washing with PBS, the cells were scraped into ice-cold PBS and pelleted. The pellets were then lysed with RIPA buffer supplemented with proteinase inhibitors according to the manufacturer’s recommendation (1 h on ice), and the extracts were cleared with centrifugation (15,000 rpm/10 min). Proteins containing supernatants were combined with 2× Laemmli loading buffer (125 mM Tris–HCl, 20% glycerol, 10% 2-mercaptoethanol, 4% SDS, and 0.004% bromophenol blue) and heated at 95 °C for 10 min. 4–20% SDS-PAGE (Mini-PROTEAN TGX Precast Gel) was used to resolve the proteins. After transferring to the PVDF membrane, the proteins were detected using appropriate antibodies: BiP (C50B12) Rabbit (Cell Signaling), Calnexin (C5C9) Rabbit (Cell Signaling), Ero1-Lα Antibody 3264 (Cell Signaling), IRE1α (14C10) Rabbit (Cell Signaling), PDI (C81H6) Rabbit (Cell Signaling), CHOP (L63F7) Mouse (Cell Signaling), PERK (D11A8) Rabbit (Cell Signaling), GAPDH Mouse (Sigma-Aldrich), Anti-rabbit IgG, HRP-linked Antibody (Cell Signaling), and Anti-mouse IgG, HRP-linked Antibody (Cell Signaling). SignalFire ECL Reagent (A + B) was used as a substrate for HRP, and the luminescence was recorded with Amersham Imager 680. The densities in the images were assessed with Aida image analysis software.

### Phototoxicity Testing

The phototoxic activity of Os complexes was determined against human cervix adenocarcinoma cells (HeLa), oesophageal carcinoma (OE33), and skin melanoma cells (A375). Cells were seeded on 96-well tissue culture plates at a density of 5 × 10^3^ cells/well (HeLa, A375) or 5.5 × 10^3^ cells/well (OE33) in 100 μL of medium and cultured under normoxic (21% O_2_, 5% CO_2_) or hypoxic (3% O_2_, 5% CO_2_) conditions in a humidified incubator. After overnight incubation, the medium was removed, the tested compound diluted in Earle’s balanced salt solution (EBSS) was added, and cells were incubated for 1 h in the dark. After the incubation period, the cells were irradiated for 1 h (λ_max_ = 533 nm) or left in the dark. Subsequently, EBSS was removed, and cells were returned to the incubator in a complete drug-free culture medium. The metabolic activity of the cells was determined 70 h after irradiation using a standard MTT assay (absorbance measured at 570 nm). The IC_50_ values were obtained from concentration–response cell survival curves [cell survival (%) versus drug concentration (μM)]. All experiments were carried out in triplicate, and at least three independent experiments were performed.

## Supporting Information Available

Synthetic schemes of ligand dpq and diamine A; characterization of complexes; ^1^H NMR aggregation experiments; photophysical properties; X-ray diffraction; stability in cell culture medium; photostability with white light irradiation; singlet oxygen production; and biological experiments (PDF)
