# Supplementary material for: Cyclometalated Benzimidazole Osmium(II) Complexes with Antiproliferative Activity in Cancer Cells Disrupt Calcium Homeostasis
Source: Inorg Chem. 2023 Apr 11;62(16):6474–87. doi: 10.1021/acs.inorgchem.3c00501 (PMC10131226; doi:10.1021/acs.inorgchem.3c00501)
Supplement: Supplementary file 1 — ic3c00501_si_002.pdf [file ic3c00501_si_002.pdf]

Supporting Information

for

Cyclometalated benzimidazole osmium(II) complexes with antiproliferative activity in cancer cells disrupt calcium homeostasis

Alba Hernández-García[a], Lenka Markova[b], María Dolores Santana[a], Jitka Pracharova[b,c], Delia Bautista[d], Hana Kostrhunova[b], Vojtech Novohradsky[b], Viktor Brabec[b,c], José Ruiz[a*], Jana Kasparkova[b*]

[a] *Departamento de Química Inorgánica, Universidad de Murcia, and Murcia BioHealth Research Institute (IMIB-Arrixaca), E-30071 Murcia, Spain.*
[b] *Czech Academy of Sciences, Institute of Biophysics, CZ-61200 Brno, Czech Republic.*
[c] *Department of Biophysics, Faculty of Science, Palacky University in Olomouc, CZ-78371 Olomouc, Czech Republic.*
[d] *SUIC-ACTI, Universidad de Murcia, E-30071 Murcia, Spain.*

Table of contents

1. Synthetic schemes of ligand dpq and diamine A S2
2. Characterization of complexes S3
   2.1 Nuclear Magnetic Resonance (NMR) S3
   2.2 Mass spectrometry S11
3. ^{1}H-NMR aggregation xperiments S17
4. Photophysical properties S18
5. X-ray iffraction S18
6. Stability in cell culture medium S20
7. Photostability with white light irradiation S22
8. Singlet oxygen production S23
9. Biological experiments S23

1. Synthetic schemes of ligand dpq and diamine A

The diamine **A** (Scheme S1) was obtained as previously described (J. Med. Chem. 2015, 58, 7310–7327).

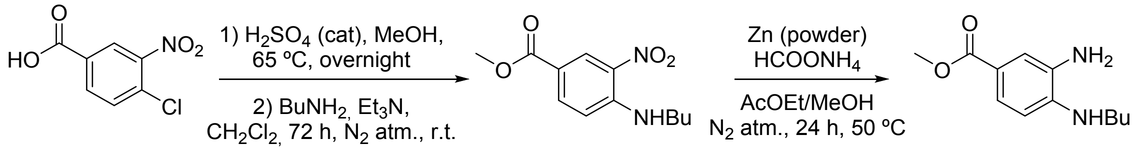

A

Scheme S1. Synthetic scheme to synthesize the diamine precursor **A**.

The N^N ligand **dpq** was synthesized as previously reported (Dalton Trans. 2016, 45 1,6366–16378; https://doi.org/10.1039/C6DT02416E).

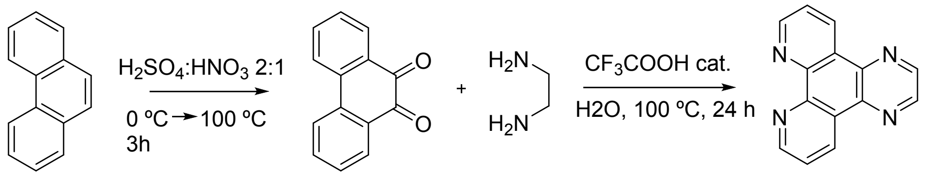

dpq

Scheme S2. The synthesis procedure of **dpq**.

2. Characterization of complexes

2.1 Nuclear Magnetic Resonance (NMR)

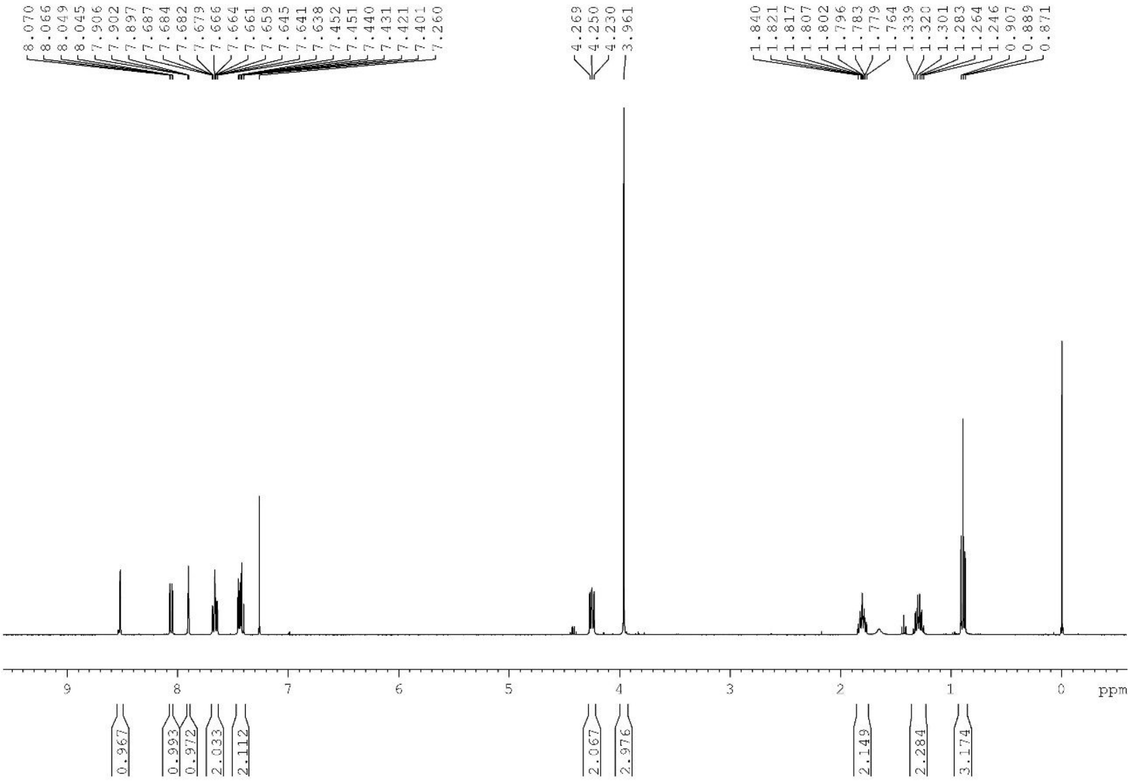

Figure S1. ^{1}H NMR of **B** in $CDCl_3$

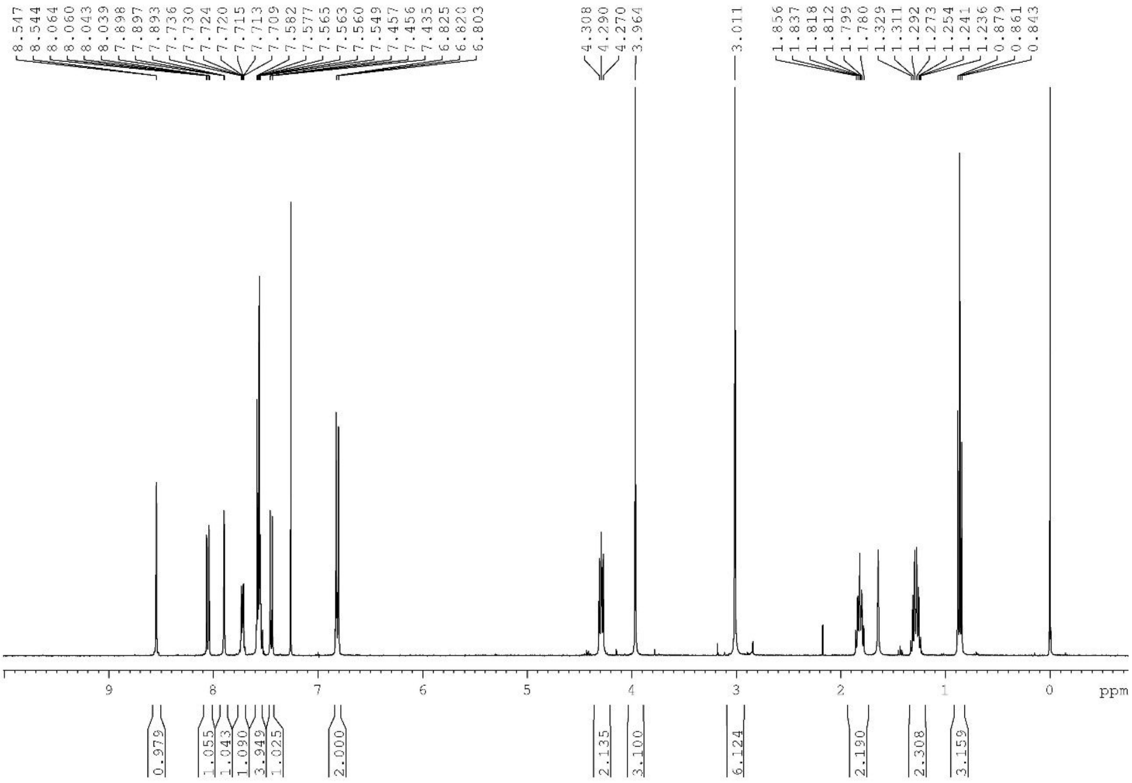

Figure S2. ^{1}H NMR of **HL2** in $CDCl_3$

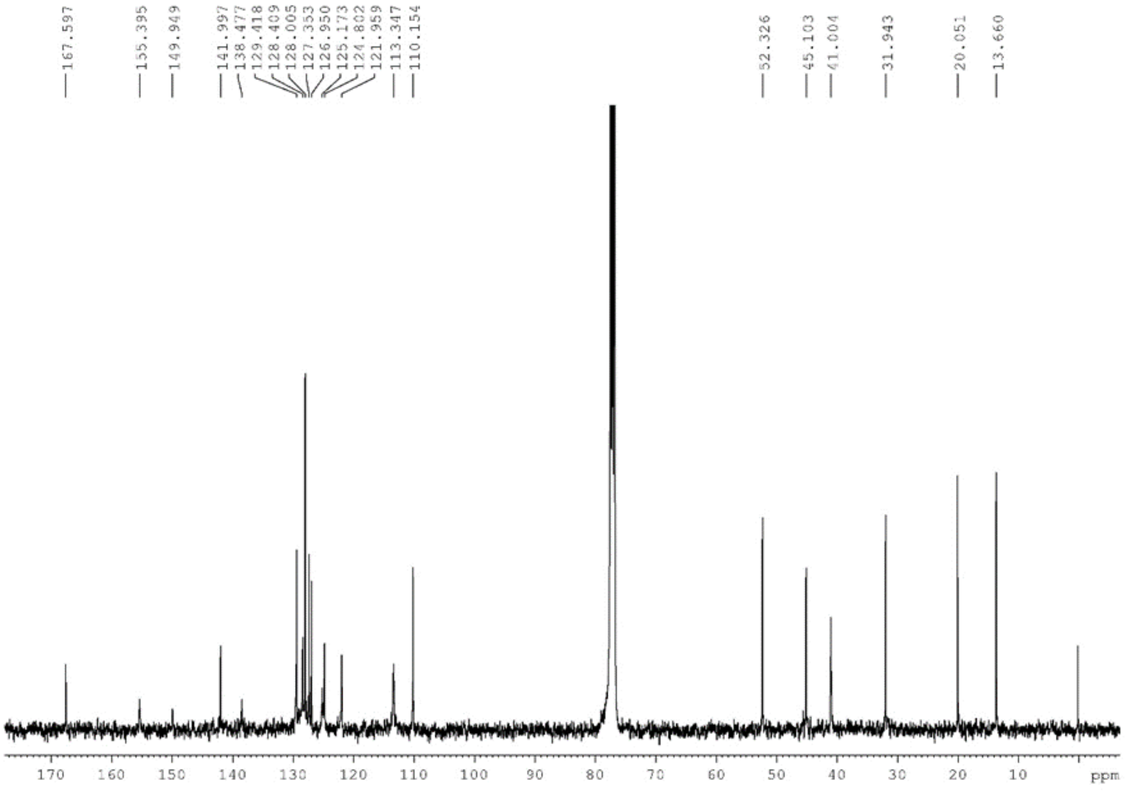

Figure S3. ^{13}C NMR of **HL2** in $CDCl_3$

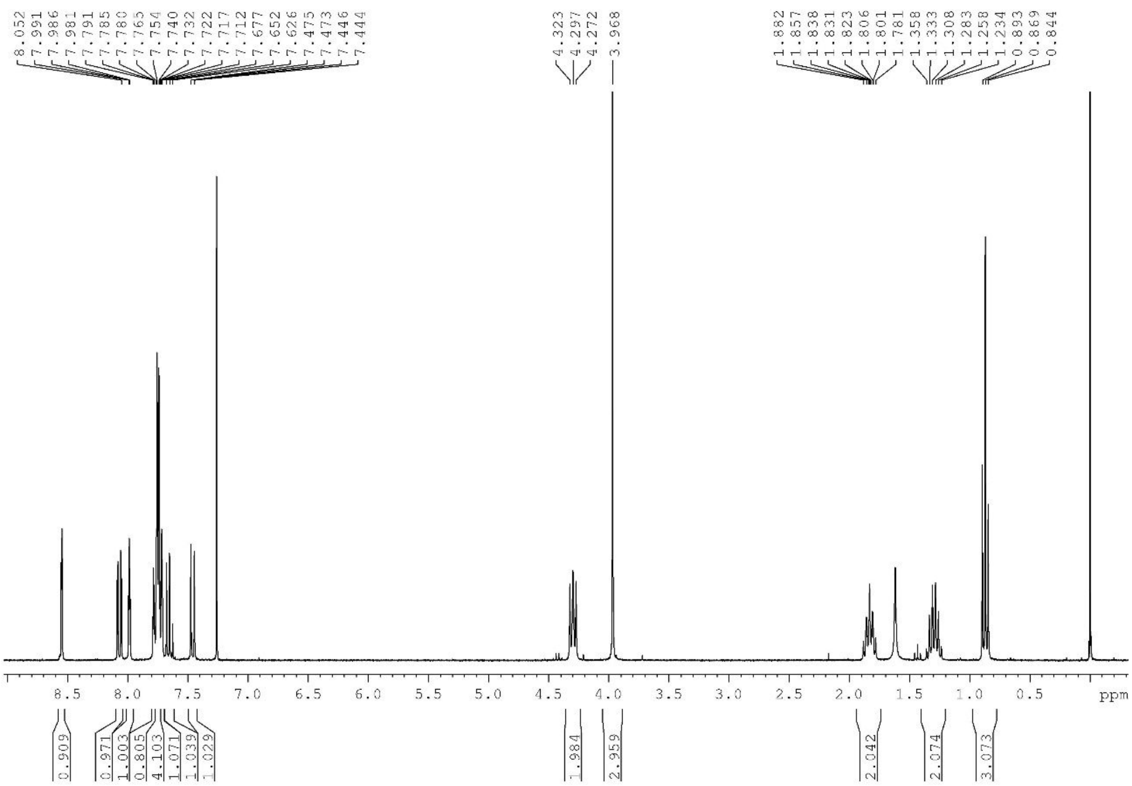

Figure S4. ^{1}H NMR of **HL3** in $CDCl_3$

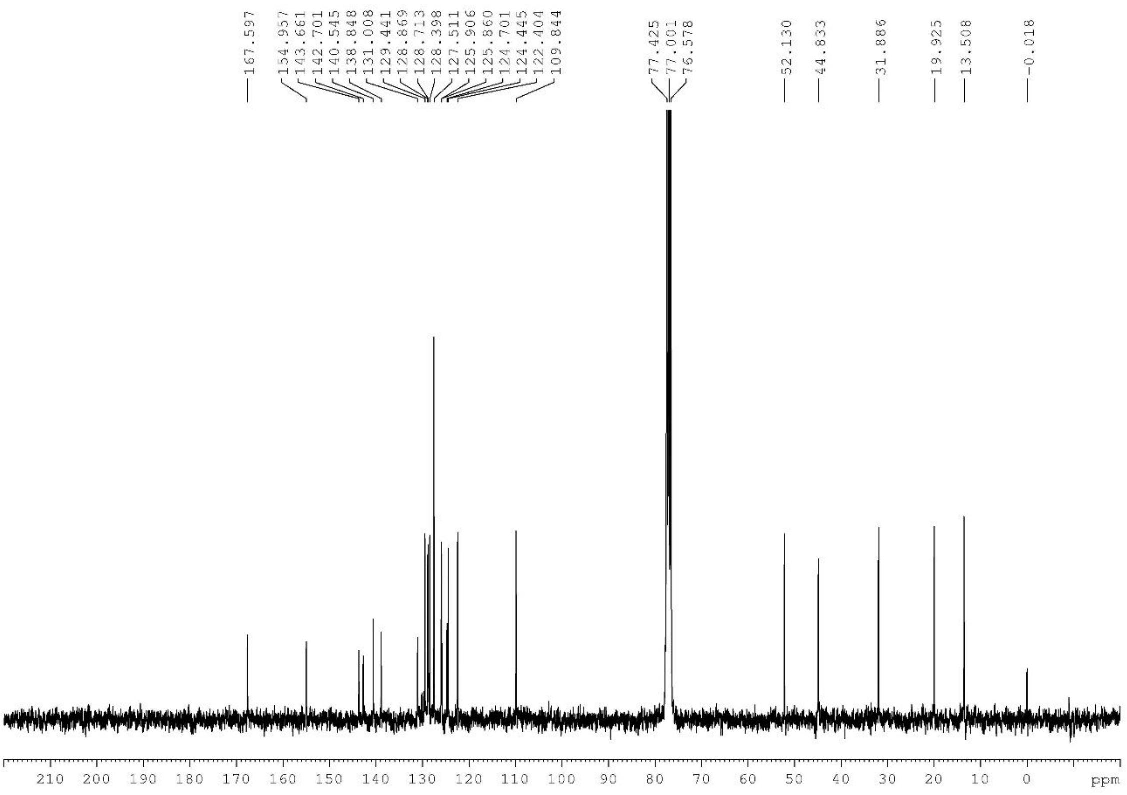

Figure S5. ^{13}C NMR of **HL3** in $CDCl_3$

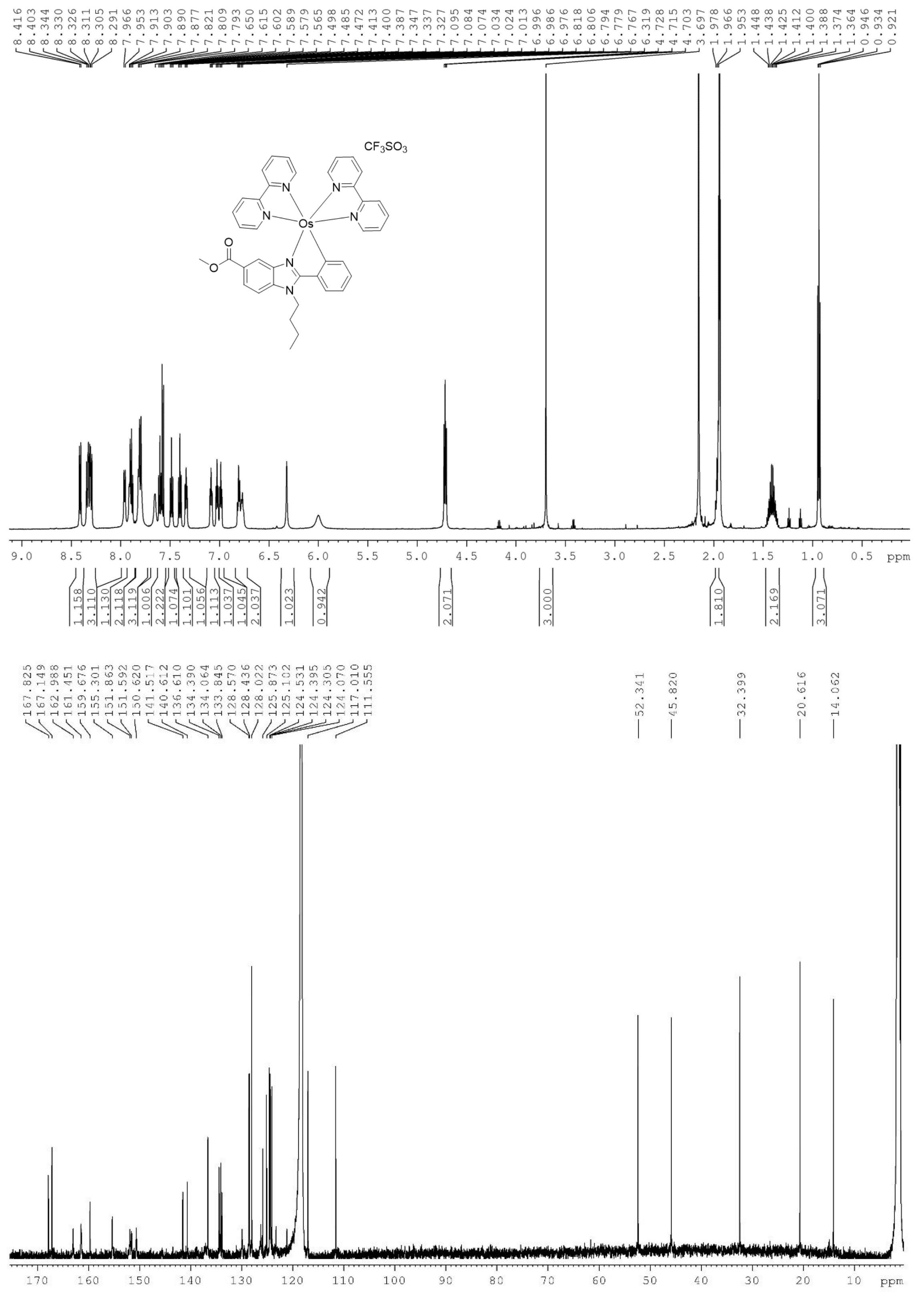

Figure S6. ^{1}H and ^{13}C NMR spectra of compound **Os1** in CD_3CN.

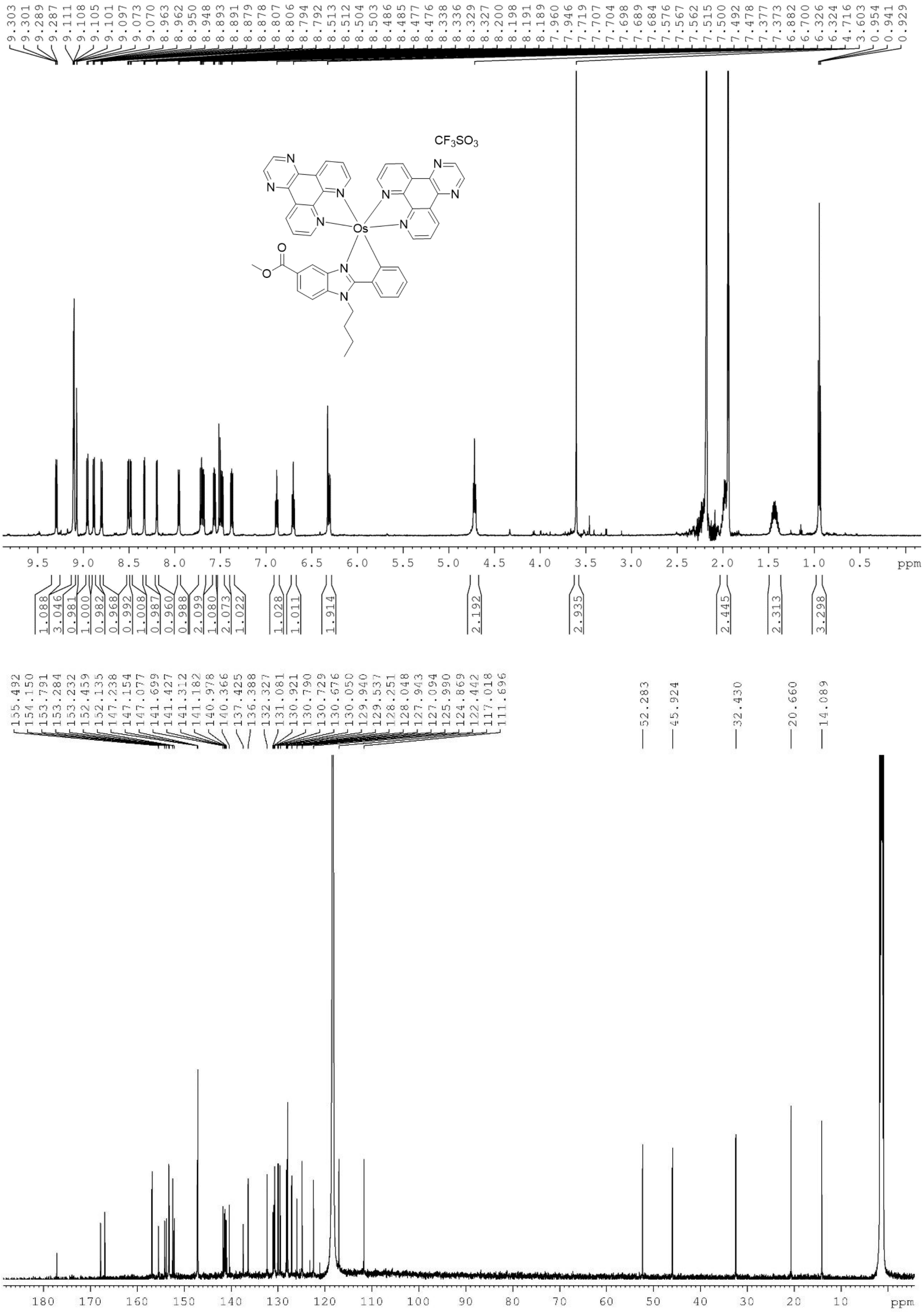

Figure S7. ^{1}H and ^{13}C NMR spectra of compound **Os2** in CD_3CN.

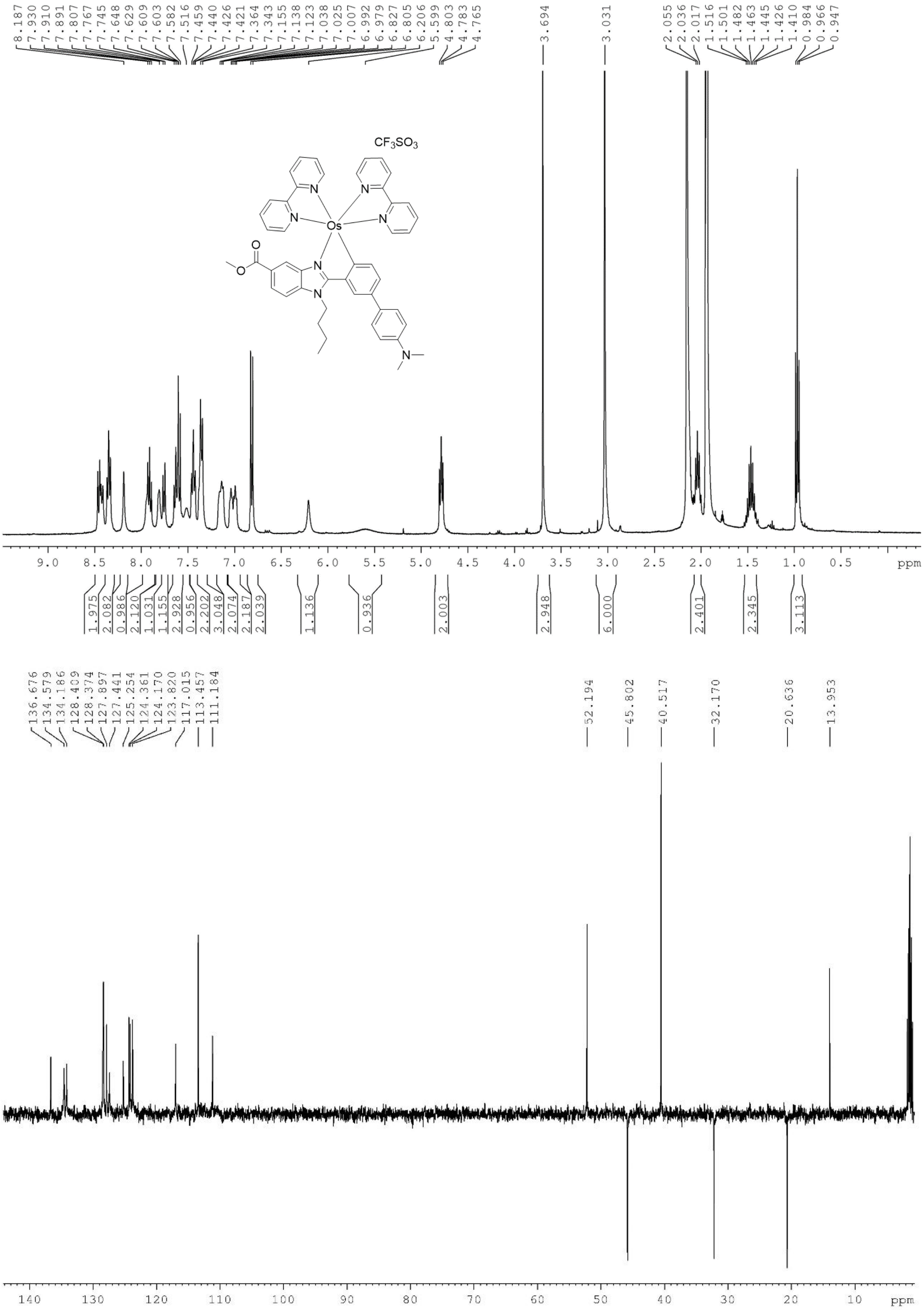

Figure S8. ^{1}H and DEPT-135 NMR spectra of compound **Os3** in CD_3CN.

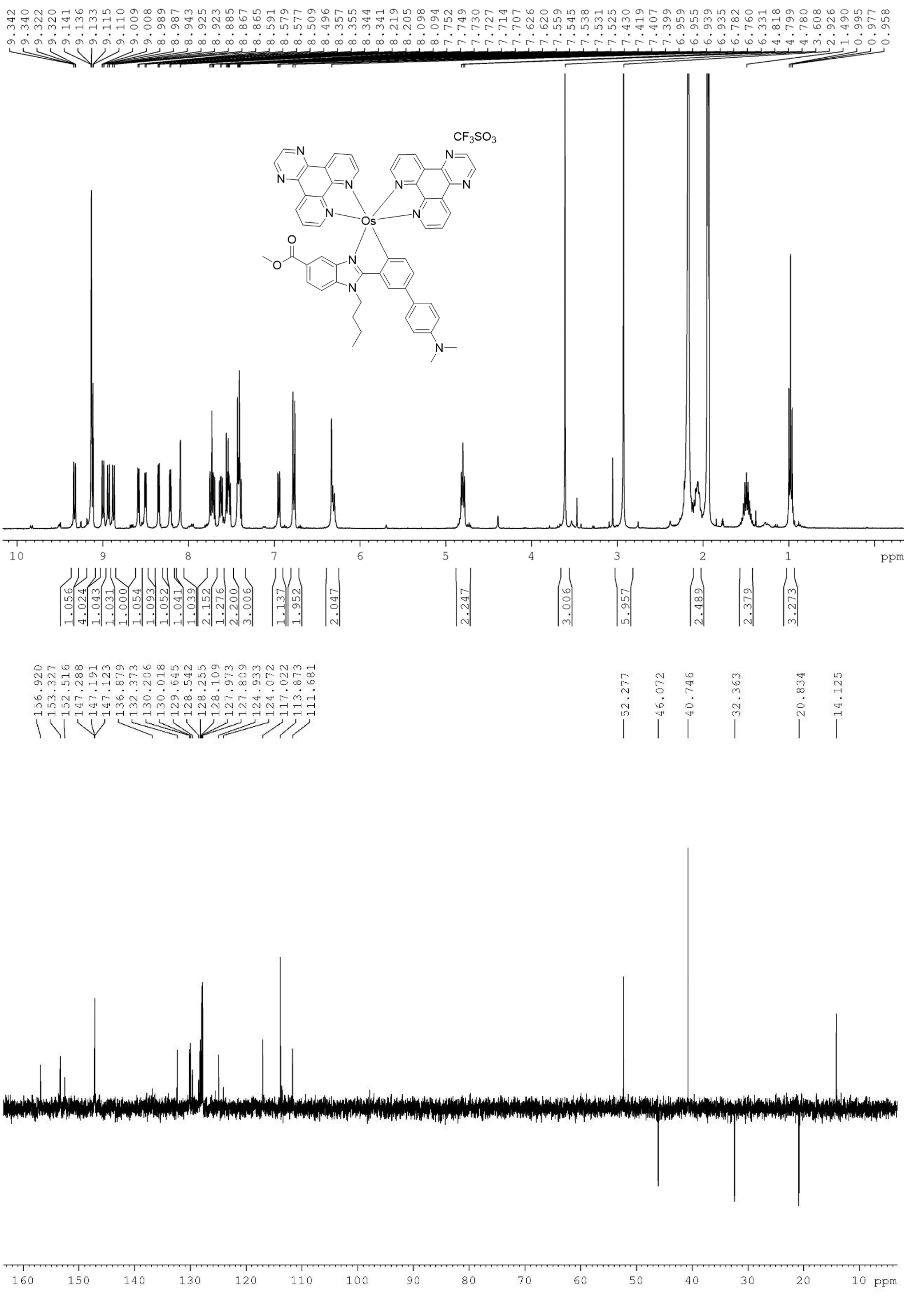

Figure S9. ^{1}H and DEPT135 NMR spectra of compound **Os4** in CD_3CN.

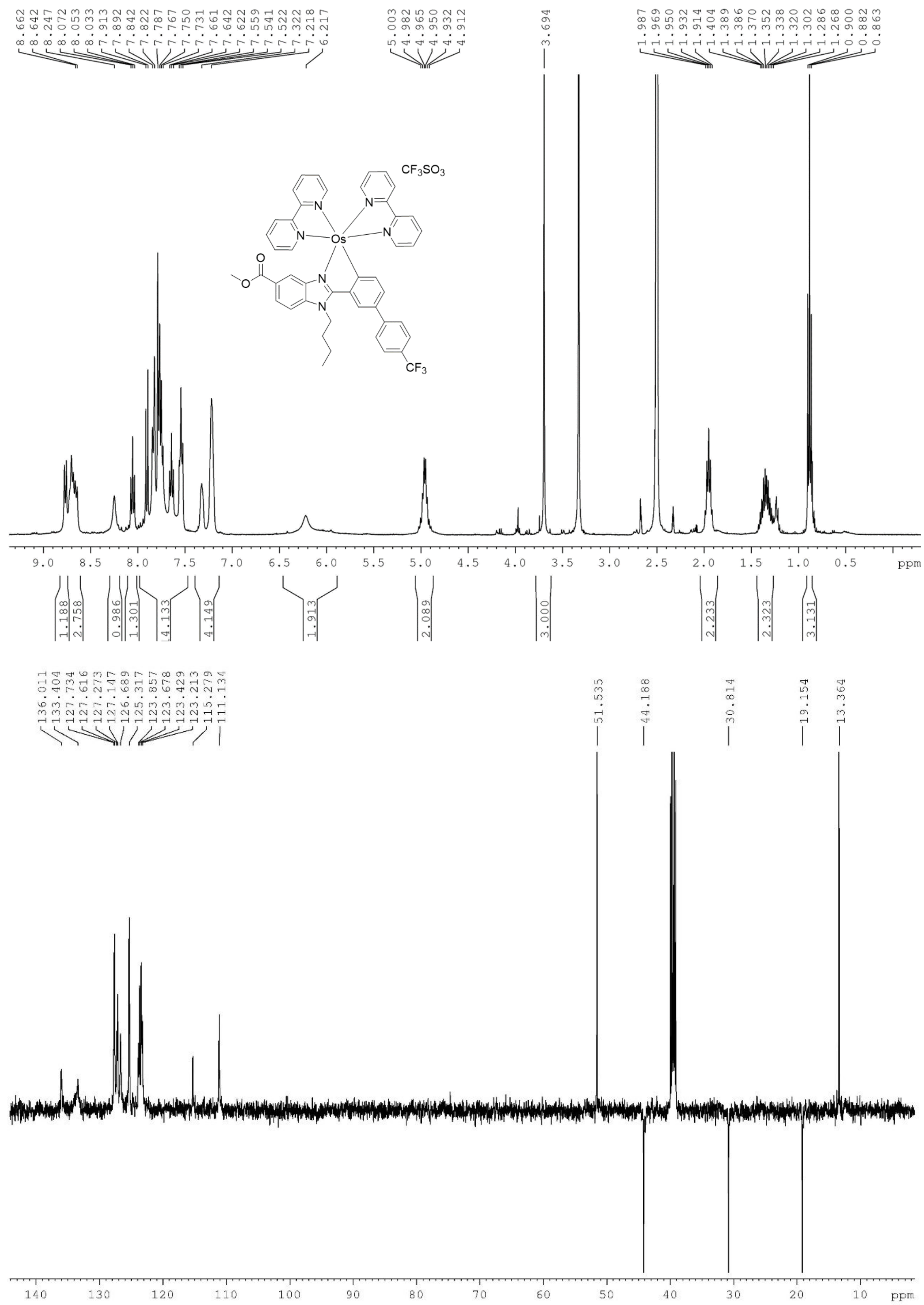

Figure S10. ^1H and DEPT-135 NMR spectra of compound **Os5** in CD_3CN.

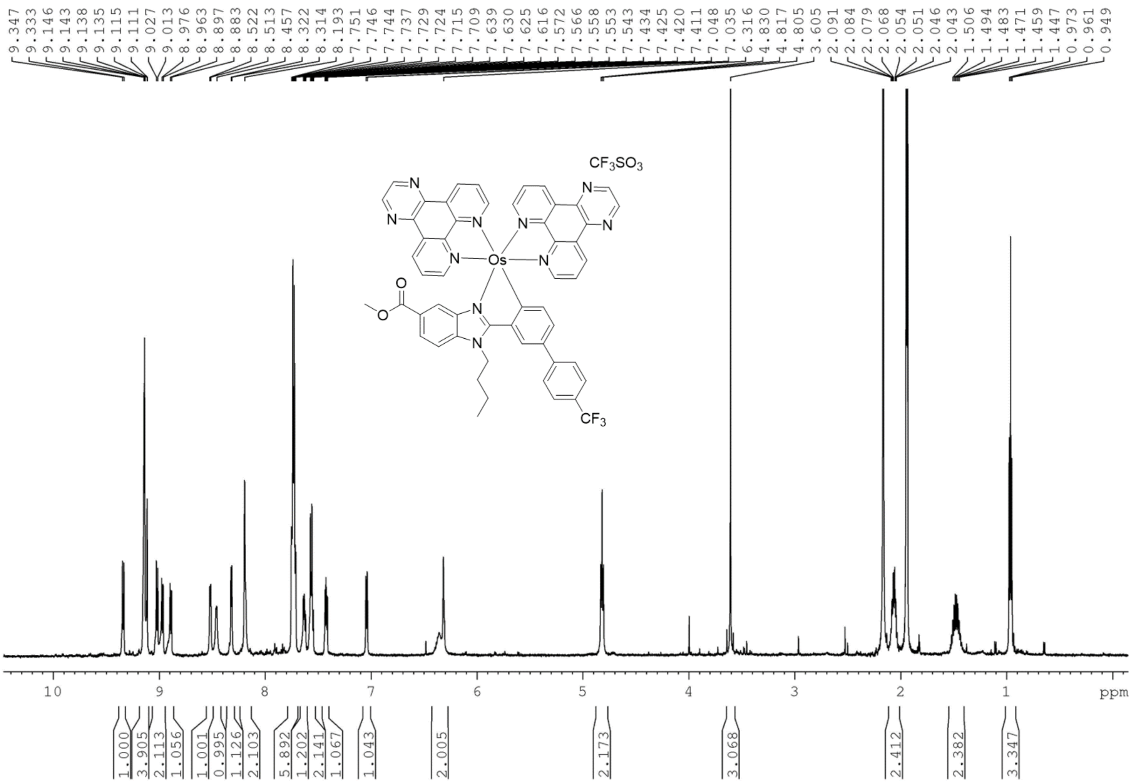

Figure S11. ^{1}H spectrum of compound **Os6** in CD_3CN.

2.2 Mass spectrometry

All complexes were characterized by mass spectrometry in a positive mode to obtain the molecular ion (M^+).

Table S1. Molecular ion of complexes **Os1-Os6**

Complex	Molecular formula of M^+	Experimental value	Theoretical value	Relative error (%)
**Os1**	$C_{39}H_{35}N_6O_2Os^+$	811.2449	811.2431	0.0022
**Os**3	$C_{47}H_{44}N_7O_2Os^+$	930.3078	930.3166	0.00095
**Os**5	$C_{46}H_{38}F_3N_6O_2Os$	955.2641	955.2618	0.00024
**Os**2	$C_{47}H_{35}N_{10}O_2Os^+$	963.2564	963.2554	0.00010
**Os**4	$C_{55}H_{44}N_{11}O_2Os^+$	1082.3292	1082.3289	0.000028
**Os6**	$C_{54}H_{38}N_{10}F_3O_2Os^+$	1107.2747	1107.2741	0.000054

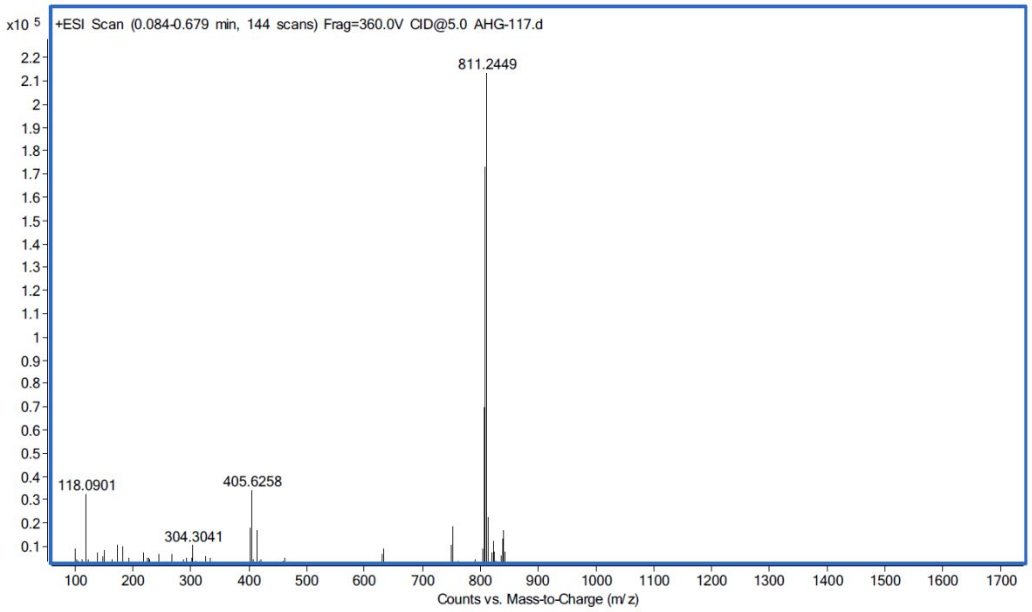

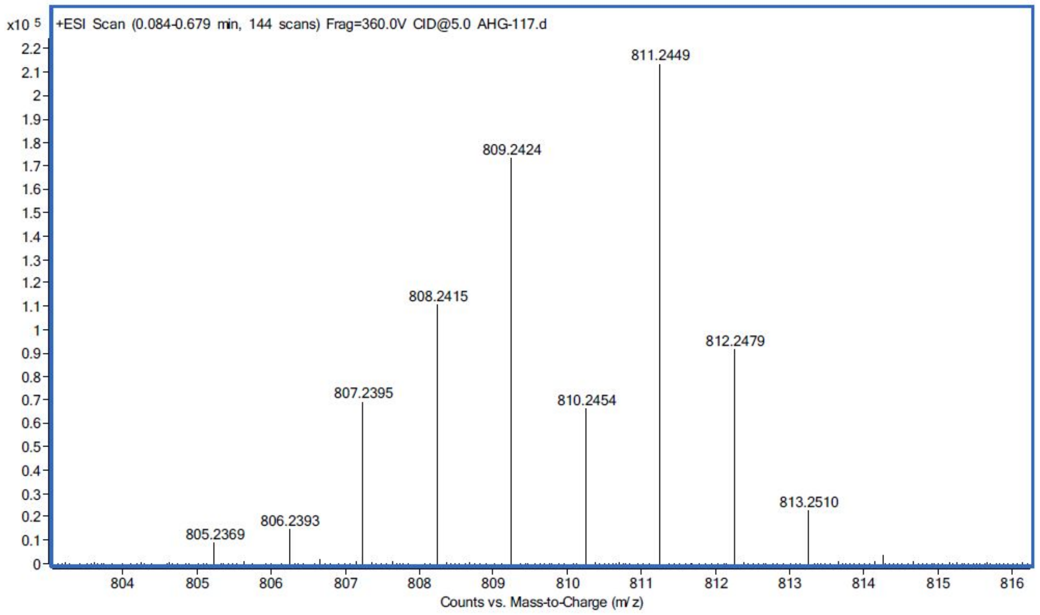

Figure S12. ESI-HRMS spectrum of complex **Os1** (positive detection mode)

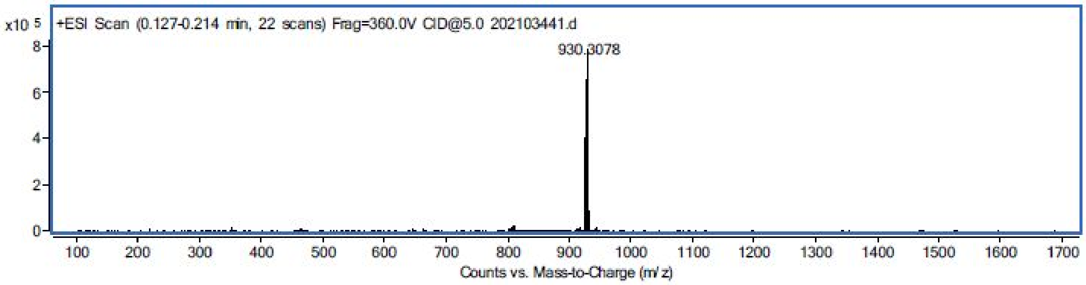

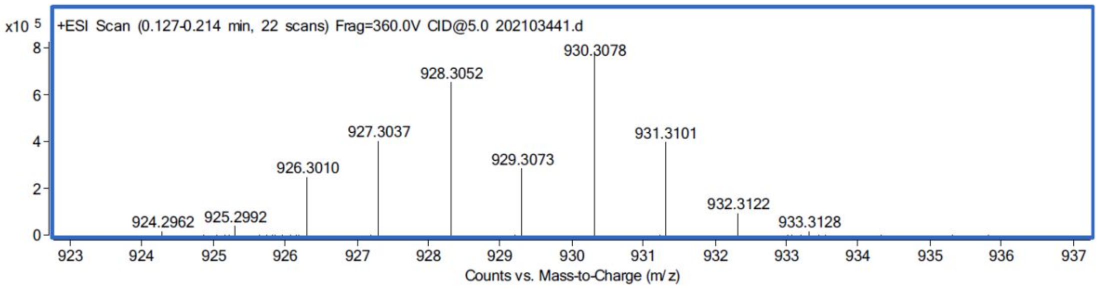

Figure S13. ESI-HRMS spectrum of complex **Os3** (positive detection mode)

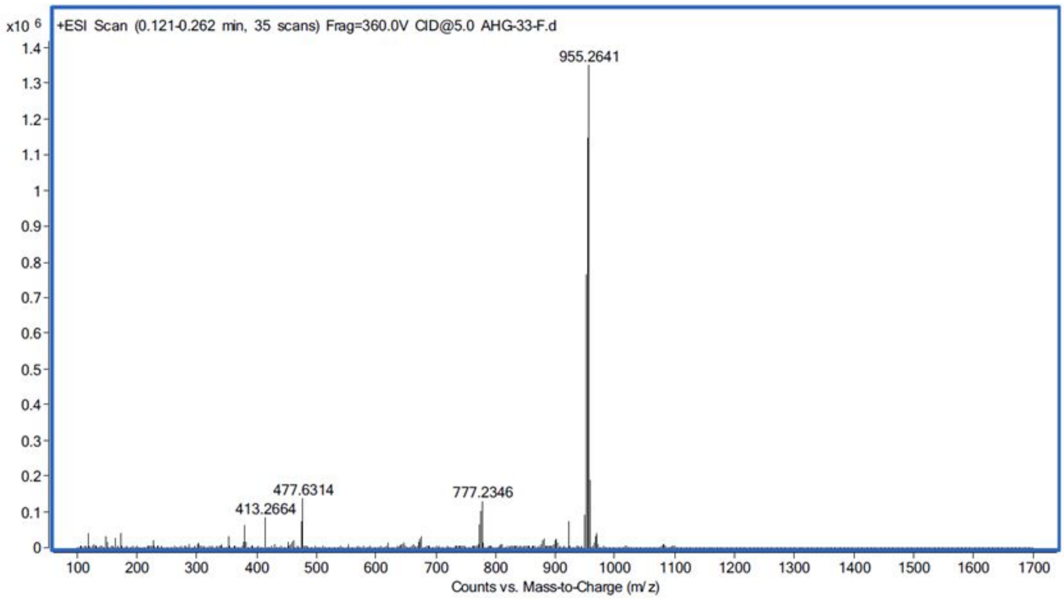

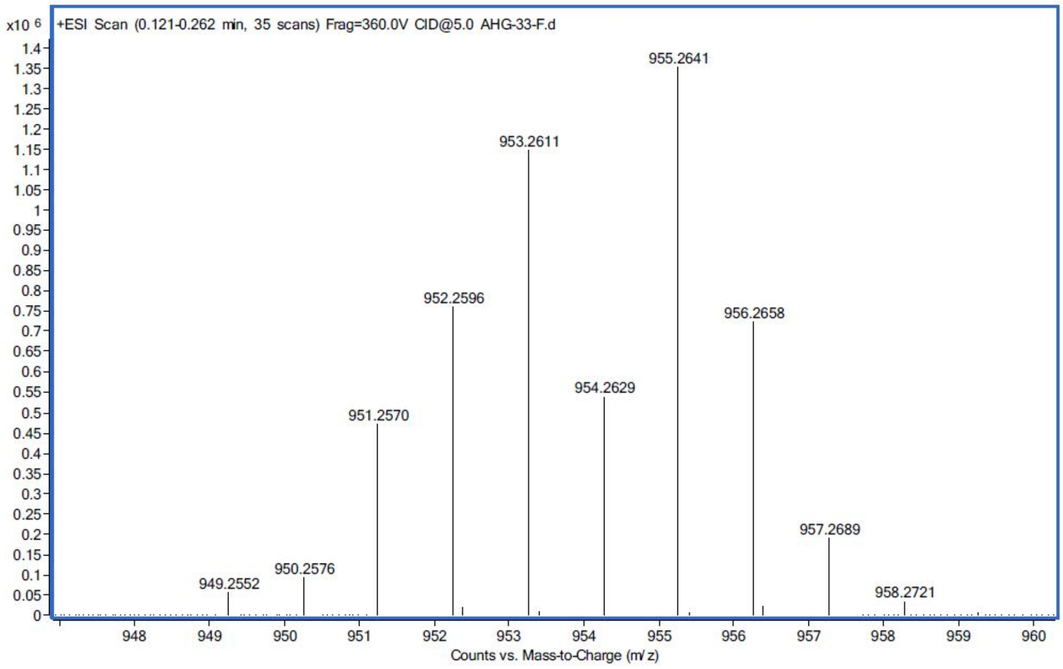

Figure S14. ESI-HRMS spectrum of complex **Os5** (positive detection mode)

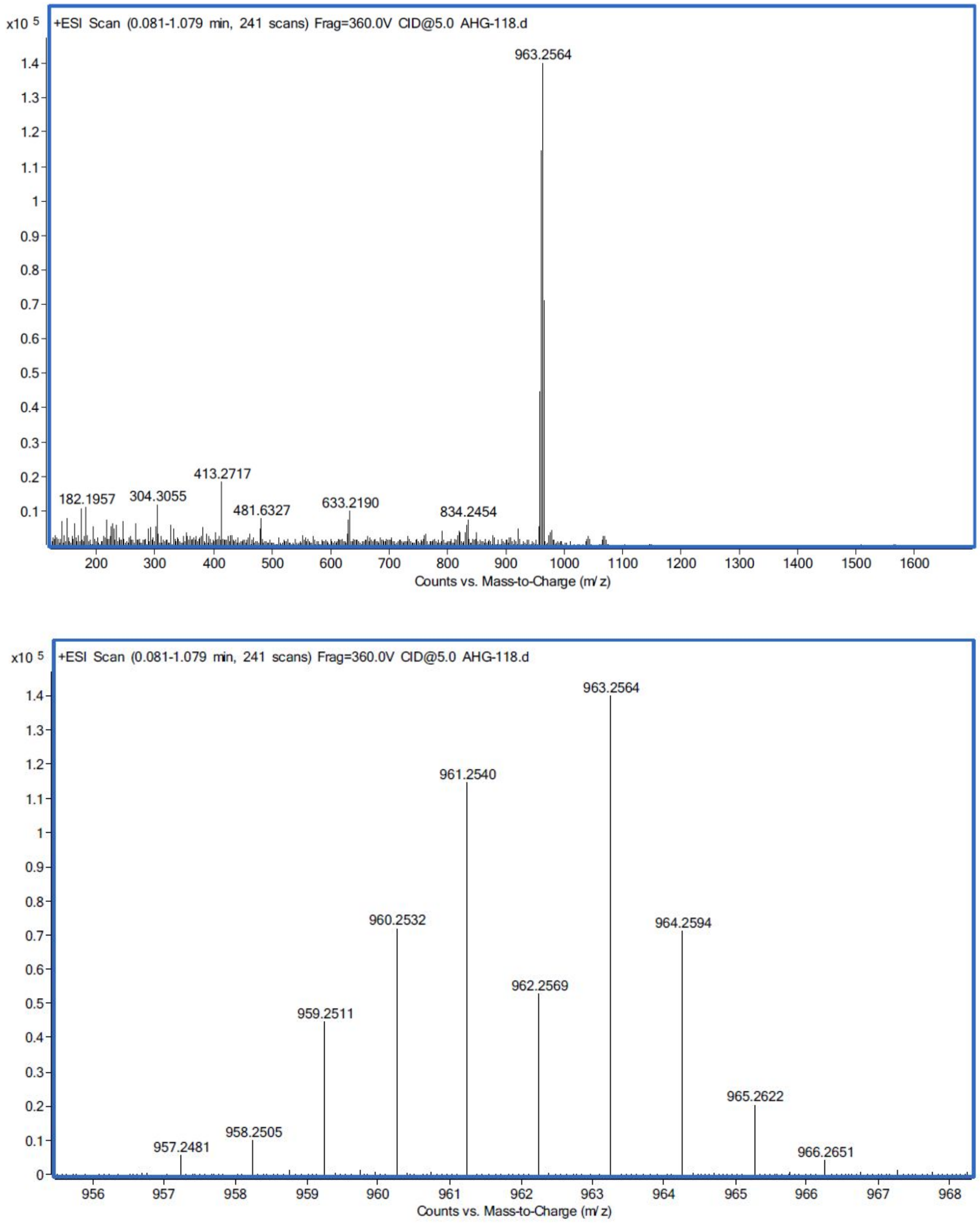

Figure S15. ESI-HRMS spectrum of complex **Os2** (positive detection mode)

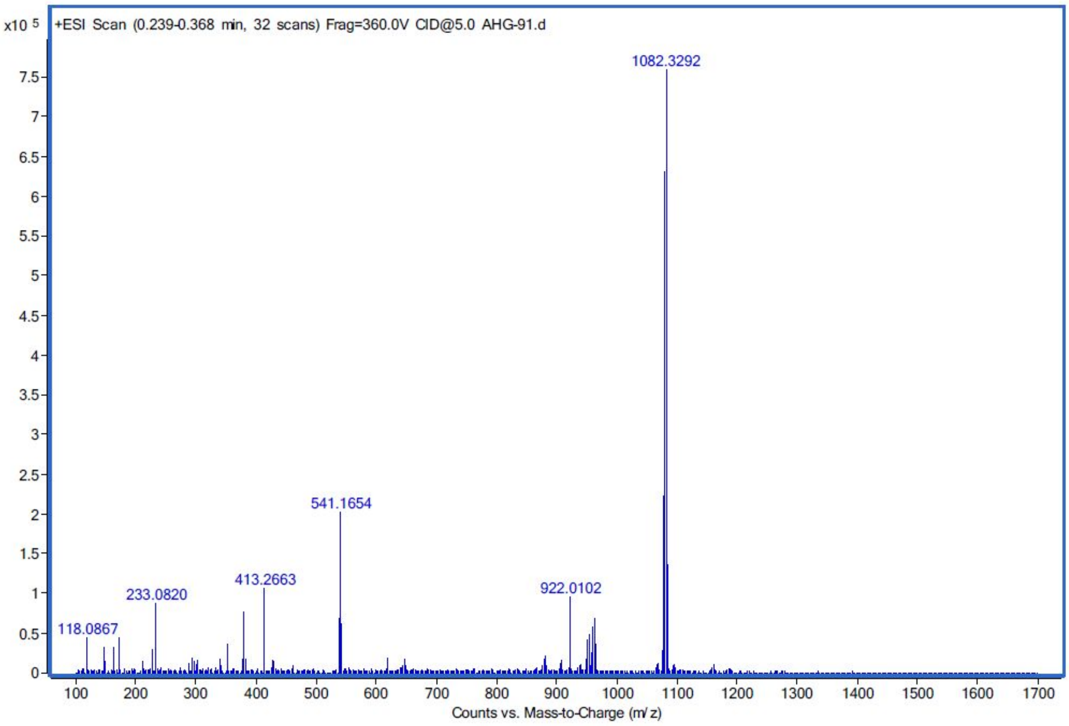

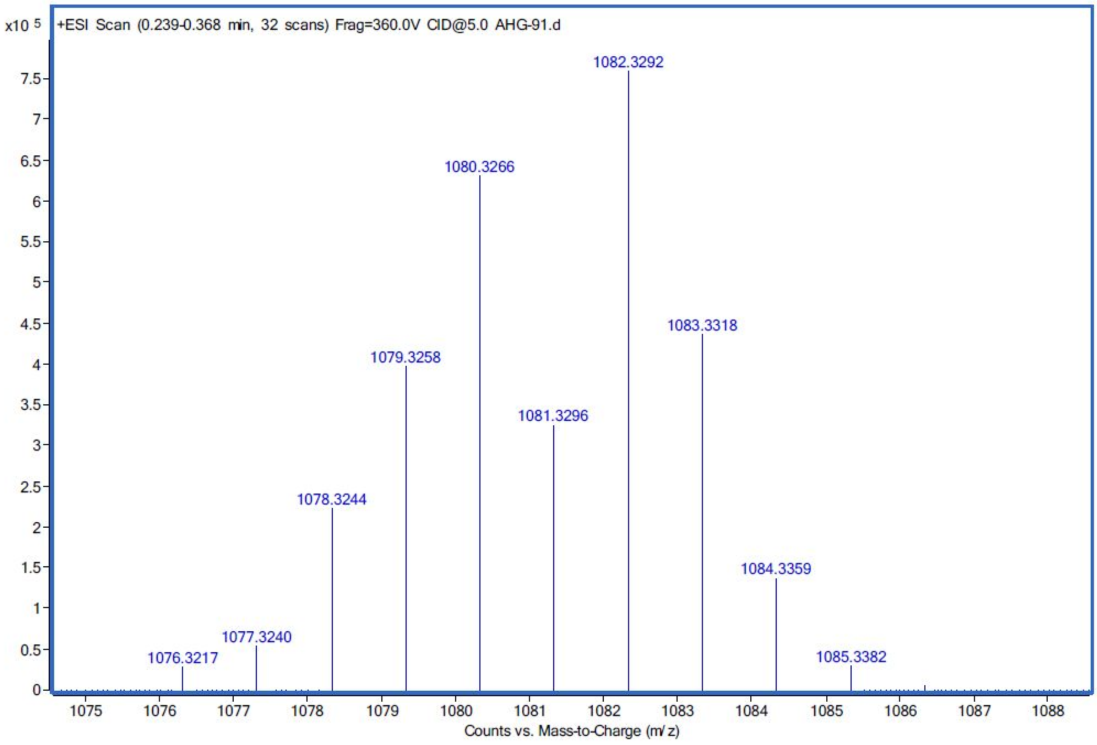

Figure S16. ESI-HRMS spectrum of complex **Os4** (positive detection mode)

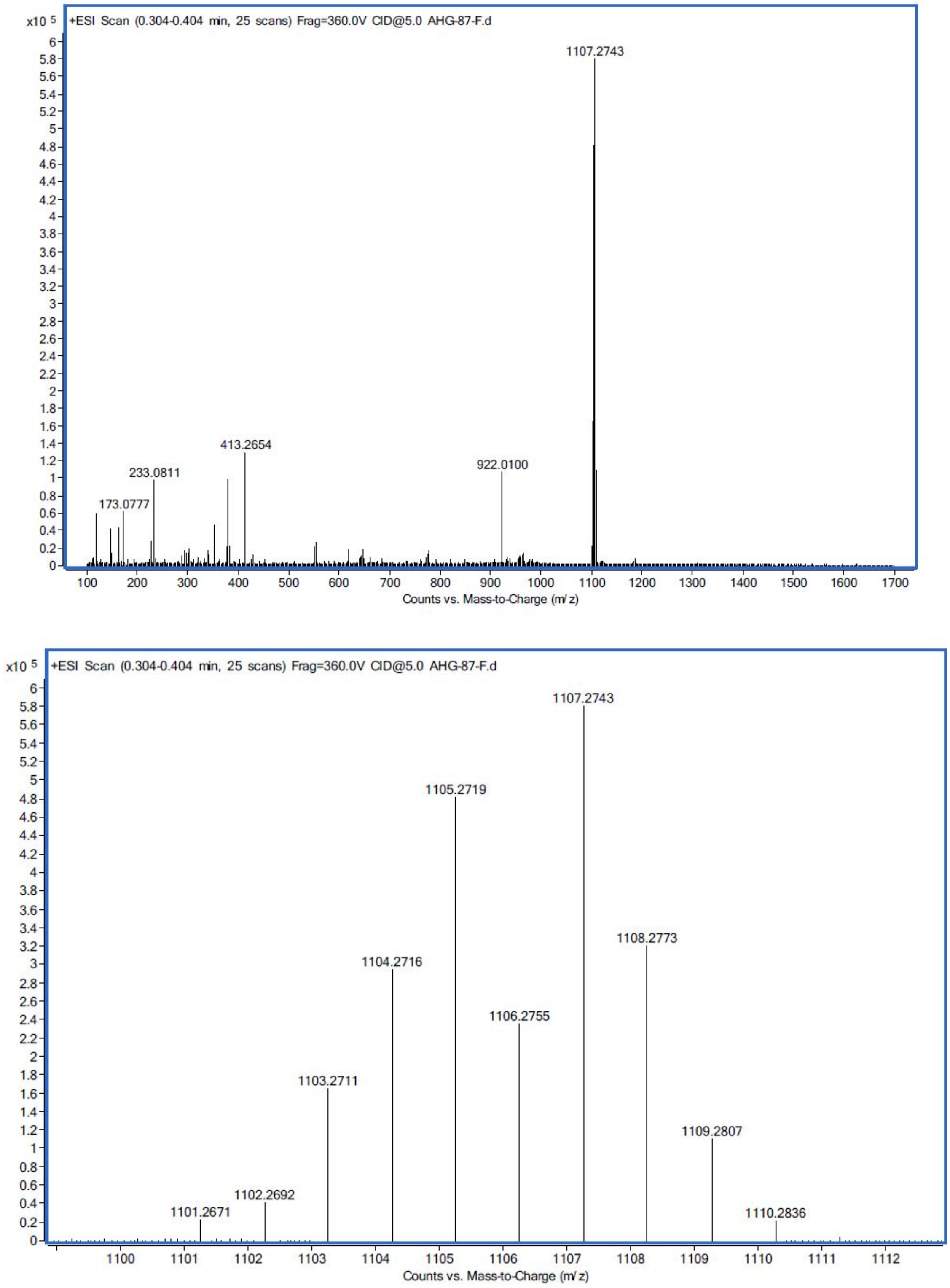

Figure S17. ESI-HRMS spectrum of complex **Os6** (positive detection mode)

3. ^{1}H-NMR aggregation experiments

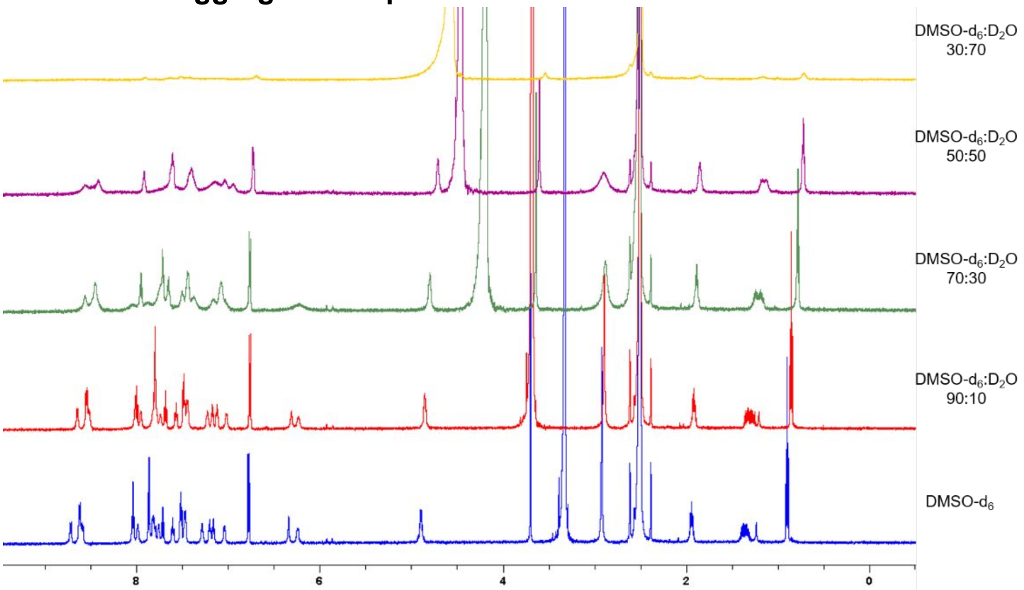

Figure S18. ^{1}NMR spectra of compound **Os3** (1mM) in deuterated DMSO and different mixtures of DMSO-d_6/D_2O

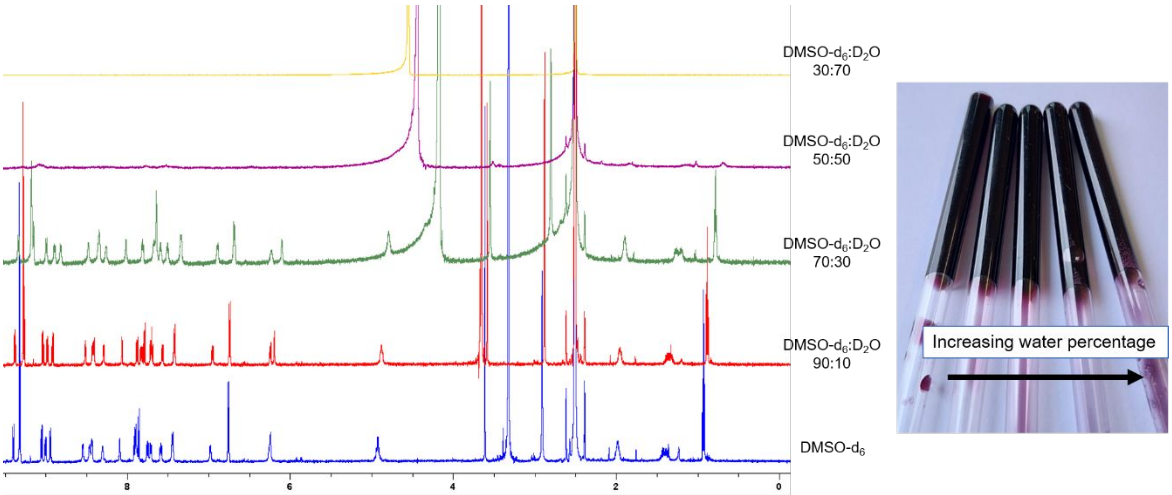

Figure S19. ^{1}NMR spectra of compound **Os4** (1mM) in deuterated DMSO and different mixtures of DMSO-d_6/D_2O.

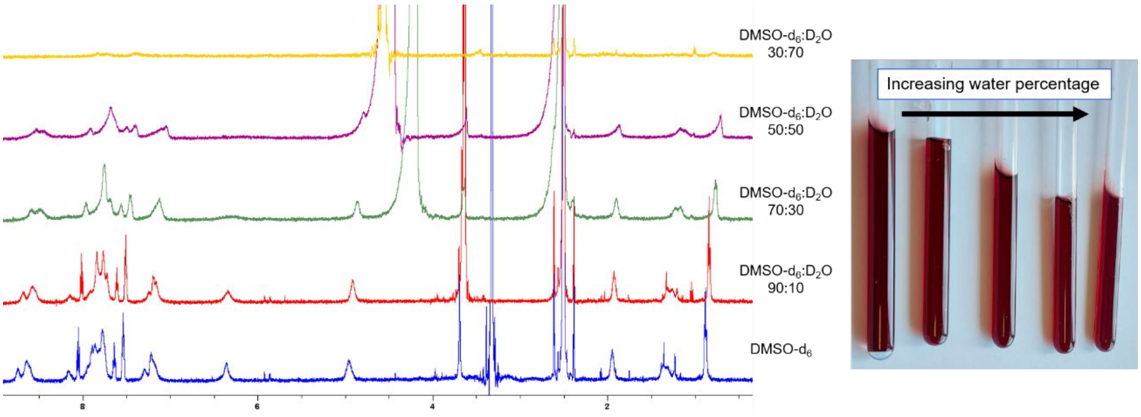

Figure S20. ^{1}NMR spectra of compound **Os5** (1mM) in deuterated DMSO and different mixtures of DMSO-d_6/D_2O.

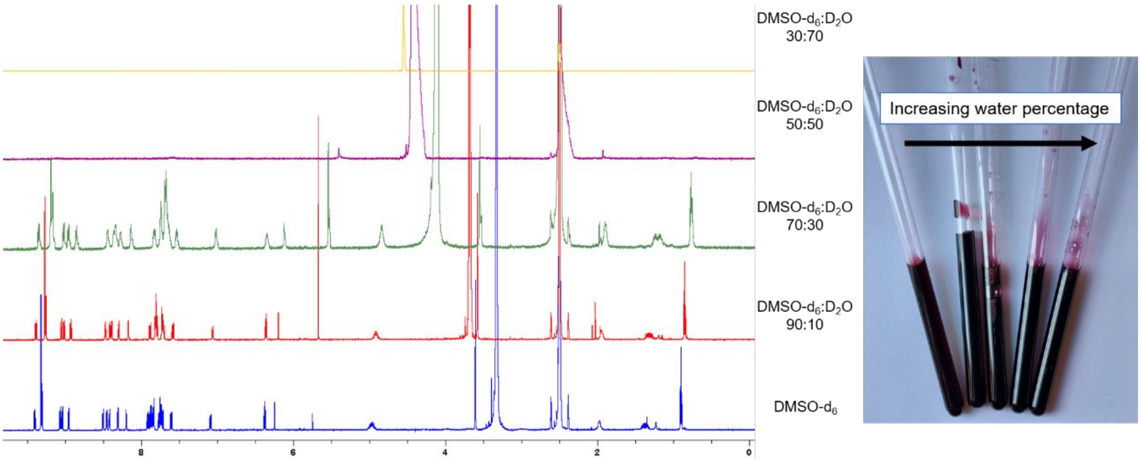

Figure S21. [1]NMR spectra of compound **Os6** (1mM) in deuterated DMSO and different mixtures of DMSO-d_6/D_2O.

4. Photophysical properties

The UV-Visible spectra of complexes were recorded in aerated CH_3CN solutions with a concentration of 10 μM.

Table S2. Photophysical properties of complexes **Os1- Os6**

Complex	λ (nm) (ε, $M^{-1}cm^{-1}$)
**Os1**	562 (10220), 296 (60490), 249 (51520)
**Os2**	554 (13020), 288 (49920), 258 (88870)
**Os3**	567 (7590), 431 (9160), 360 (16250), 298 (55920), 249 (38270)
**Os4**	560 (15230), 299 (70410), 258 (104710)
**Os5**	563 (10060), 501 (9190), 361 (17210), 297 (66910), 248 (62200)
**Os6**	554 5200), 293 (61250), 258 (117140)

5. X-ray diffraction

Table S3. Crystal data and structure refinement for complex **Os3**

**Empirical formula**	$C_{48}H_{44}F_3N_7O_5OsS$
**Formula weight**	1078.16
**Temperature / K**	100(2)
**Wavelength / Å**	0.71073
**Crystal system**	Triclinic
**Space group**	P-1
**Unit cell dimensions**	
**a / Å**	13.5749(5)
**b / Å**	14.0139(5)

**c / Å**	16.0407(6)
**α / °**	84.3400(10)
**β / °**	72.0520(10)
**γ / °**	62.2910(10)
***V*/ Å^3**	2566.51(16)
Z	2
**D_{calcd} / Mg/m^3**	1.395
**Absorption coefficient / mm^{-1}**	2.584
**F(000)**	1080
**Crystal size / mm^3**	0.120 x 0.080 x 0.020
**θ range for data collection / °**	1.877 to 27.103
**Index ranges**	-17 ≤ h ≤ 17<br>-17 ≤ k ≤ 17<br>-20 ≤ l ≤ 20
**Reflections collected**	103395
**Independent reflections**	11330 [R_{int} = 0.0301]
**Completeness to θ = 25.242°**	99.9 %
**Absorption correction**	Semi-empirical from equivalents
**Max. and min. transmission**	0.7457 and 0.6632
**Refinement method**	Full-matrix least-squares on F^2
**Data / restraints / parameters**	11330 / 0 / 590
**Goodness-of-fit on F^2**	1.119
**Final R indices [I>2σ(I)]**	R_1 = 0.0205<br>wR_2 = 0.0521
**R indices (all data)**	R_1 = 0.0231<br>wR_2 = 0.0540
**Extinction coefficient**	n/a
**Largest diff. peak and hole/ e.Å^{-3}**	1.294 and -0.798

Table S4. Hydrogen bonds for complex **Os3** [Å and °]

D-H...A	d(D-H)	d(H...A)	d(D...A)	<(DHA)
**C(9)-H(9A)...F(49)#1**	0.98	2.62	3.497(4)	148.9
**C(11)-H(11A)...O(55)**	0.99	2.53	3.397(4)	146.2
**C(34)-H(34)...O(53)#2**	0.95	2.35	3.239(3)	155.2
**C(39)-H(39)...O(1)#3**	0.95	2.56	3.202(3)	125.5
**C(41)-H(41)...O(55)#4**	0.95	2.63	3.506(4)	152.7
**C(44)-H(44)...O(55)#4**	0.95	2.65	3.480(4)	145.8

**C(47)-H(47)...N(5)**	0.95	2.64	3.188(3)	117.1

Symmetry transformations used to generate equivalent atoms:

#1 x,y,z-1 #2 -x+1,-y+2,-z+1 #3 x,y-1,z #4 -x+2,-y+1,-z+1

6. Stability in cell culture medium

The stability of complexes in the cell culture medium was evaluated by UV-Visible spectra at t=0 and after 48 hours at 37 ºC. Complexes were dissolved in RPMI (5% DMSO) with 10 μM (**Os1, Os2, Os4, Os6)** and with 1μM (**Os3** and **Os5**) of concentration.

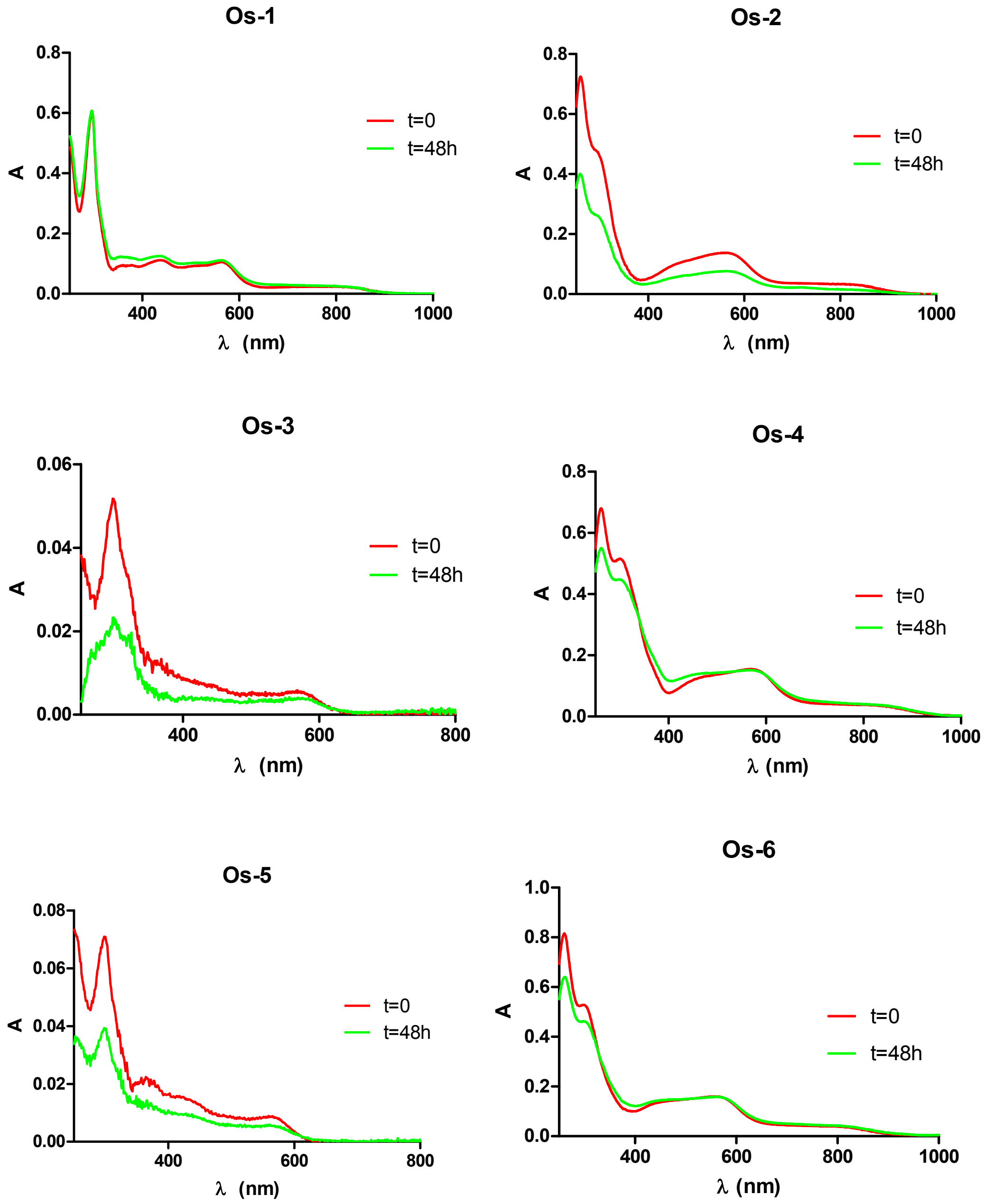

Figure S22. UV-Visible spectra of complexes **Os1-Os6** (10 μM for **Os1**, **Os2**, **Os4,** and **Os6** and 1 μM for **Os3** and **Os5**) in RPMI (5% DMSO) at t = 0 and after 48 h.

7. Photostability with white light irradiation

The complexes were dissolved in an air-saturated DMSO solution with 10 μM of concentration. To measure the photostability, osmium compounds were irradiated with white light (51 mW/cm^2) for 1.5 h. The UV-Visible spectra of complexes were recorded from 250 to 1000 nm.

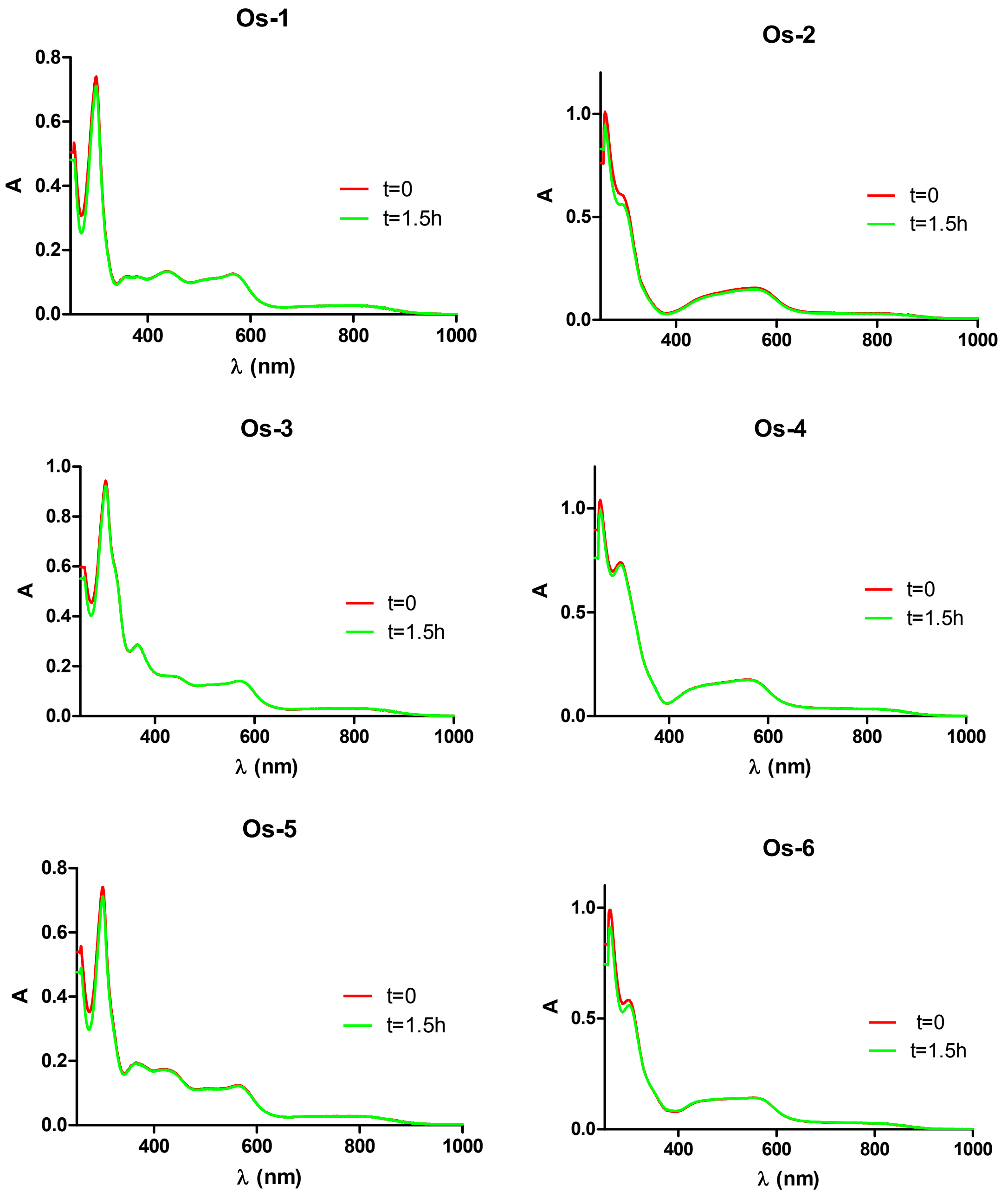

Figure S23. Time evolution of the absorbance spectra of **Os1** – **Os6** (10 μM) in an air-saturated DMSO solution upon exposure to white light (51 mW/cm^2) during 1.5 h.

8. Singlet oxygen production

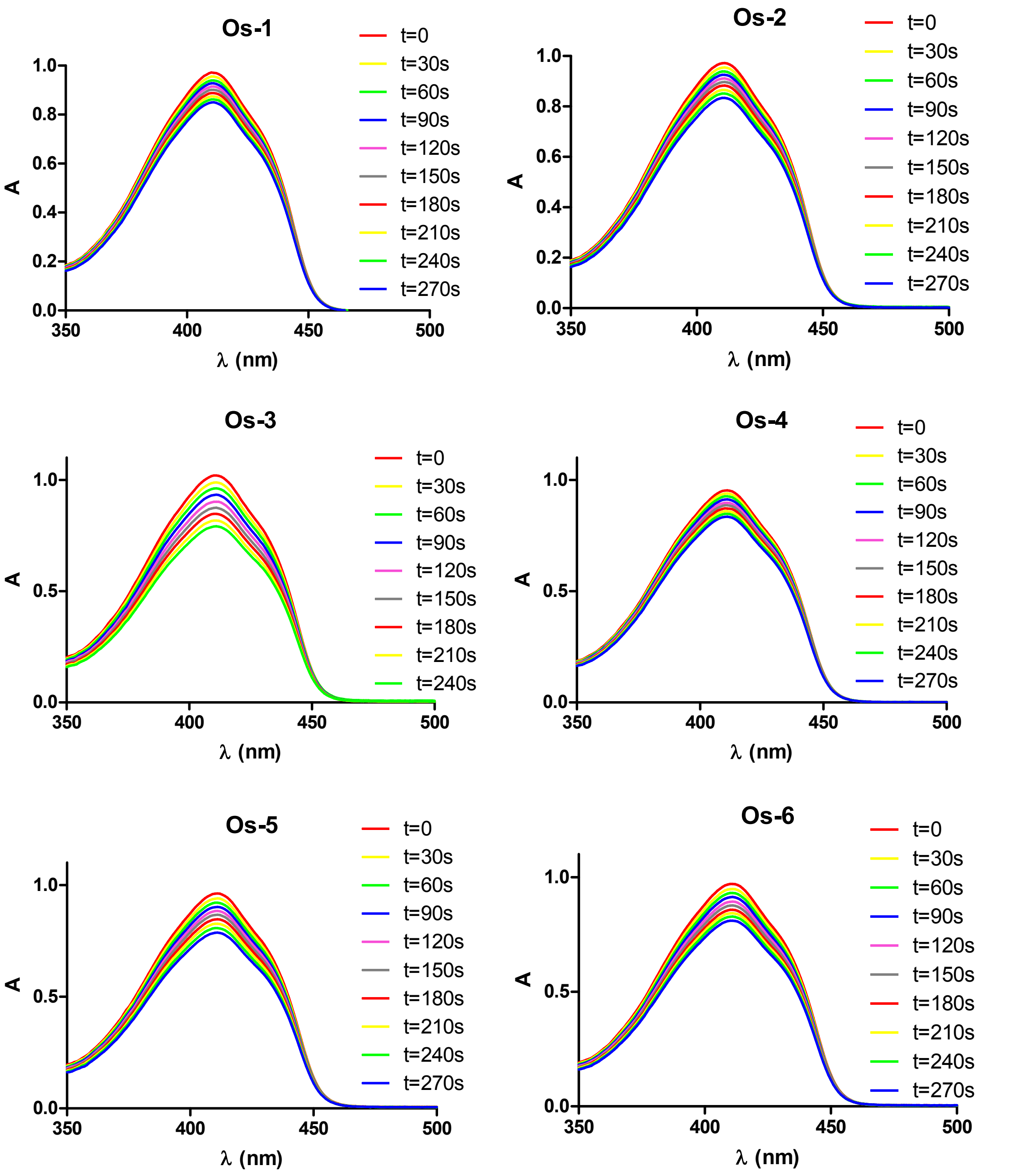

Figure S24. Changes in the absorbance measurements at 413 nm of the $^1O_2$ scavenger 1,3-diphenylisobenzofuran (DPBF) in acetonitrile solution in the presence of **Os1-Os6.** The solutions were irradiated with a laser of 520 nm (1.0 mW/cm^2) with an interval of 30 s.

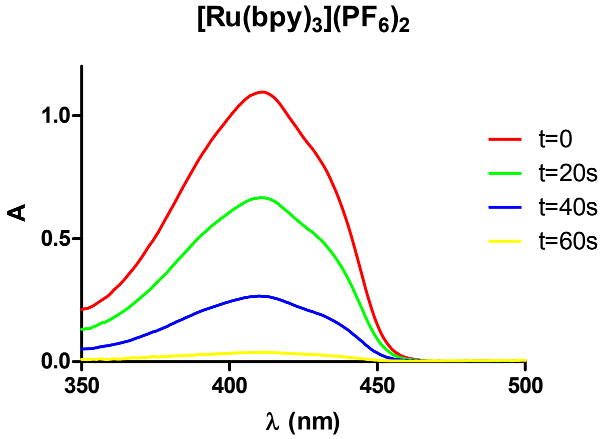

Figure S25. Changes in the absorbance measurements at 413 nm of the $^1O_2$ scavenger 1,3-diphenylisobenzofuran (DPBF) in acetonitrile solution in the presence of $[Ru(bpy)_3](PF_6)_2$ The solutions were irradiated with a laser of 520 nm (1.0 mW/cm^2) with an interval of 20 s.

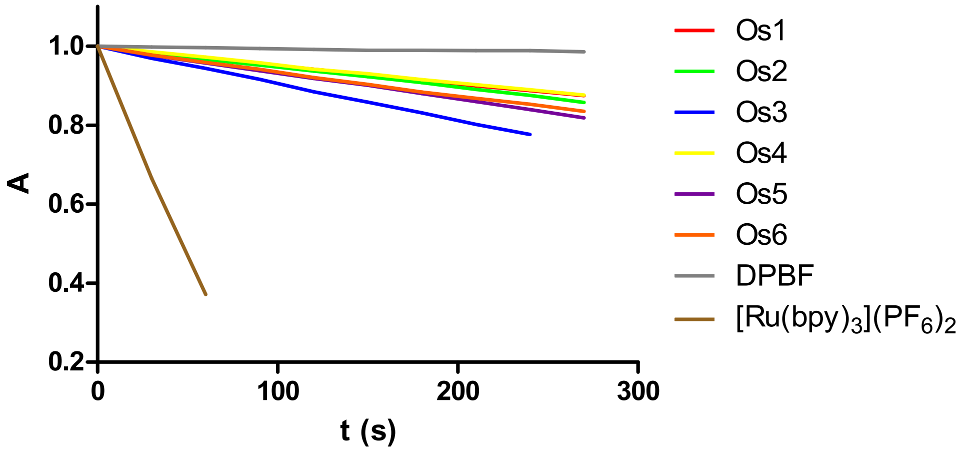

Figure S26. Plots showing the decrease in the absorbance measurements of DPBF (50 μM) at 413 nm with time on light exposure (520 nm, 1.0 mW/cm^2) in aerated acetonitrile solution in the presence of complexes **Os1-Os6** or $[Ru(bpy)_3](PF_6)_2$.

Table S5. Singlet oxygen quantum yields measured in CH_3CN.

**Complex**	**A**	m	$\Phi_{\Delta s}$
**Os1**	0.0597	-0.0005	0.01954
**Os2**	0.06256	-0.0005	0.01857
**O3**	0.06488	-0.0009	0.03214
**Os4**	0.05888	-0.0005	0.01952
**Os5**	0.0616	-0.0007	0.02643
**Os6**	0.0594	-0.0006	0.02356
$[Ru(bpy)_3](PF_6)$	0.052	-0.0126	0.57

A = absorbance; m = slopes of the linear regression; $\Phi_{\Delta s}$ = singlet oxygen quantum yield of each sample

9. Biological experiments

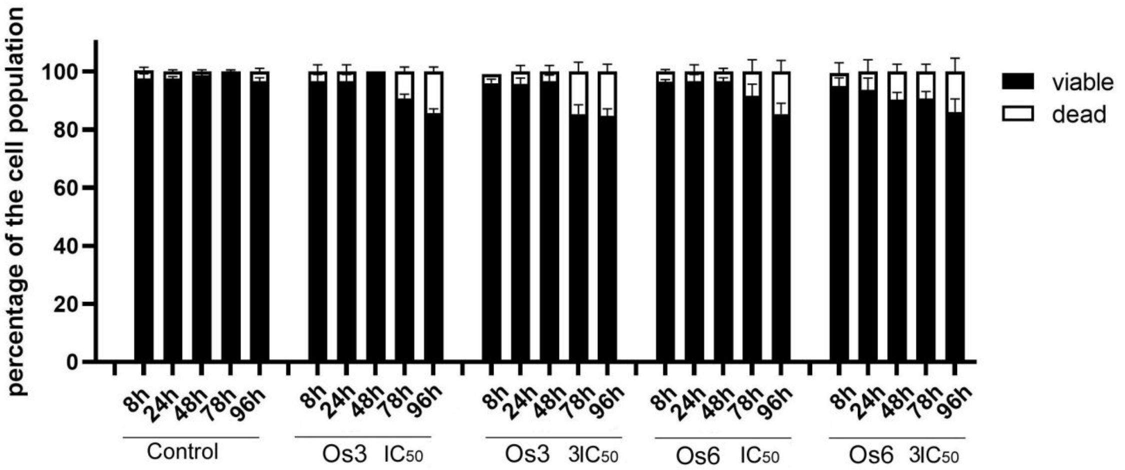

Figure S27. Percentage of viable (black) and dead (white) MBA-MB-231 cells from the total cell population assessed by the Trypan blue staining.

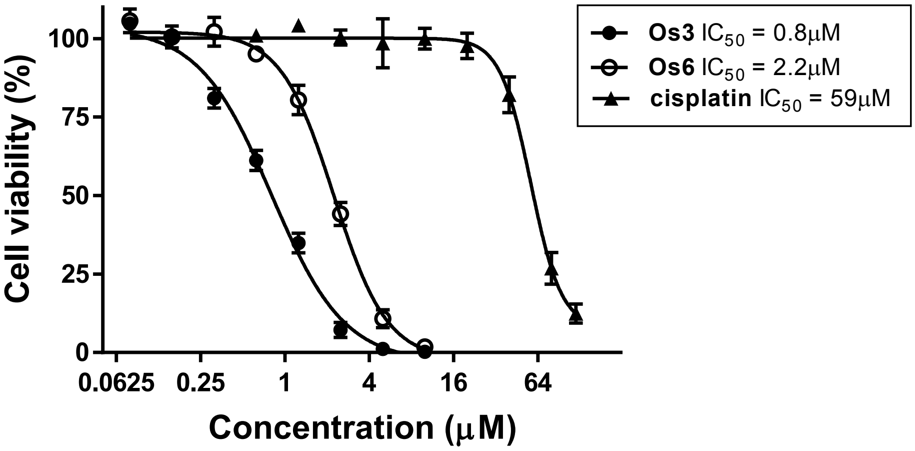

Figure S28. Sigmoidal curves of cell survival determined by CellTiter-Glo 3D reagent. Four days-old spheroids were treated with the investigated compounds **Os3**, **Os6** or cisplatin for 72 h. Treated spheroids were then transferred into black culture plate and CellTiter-Glo 3D reagent was added. After 30 min incubation the luminescence signal was measured by Tecan SPARK reader. Cell viability was normalized against vehicle treated control.

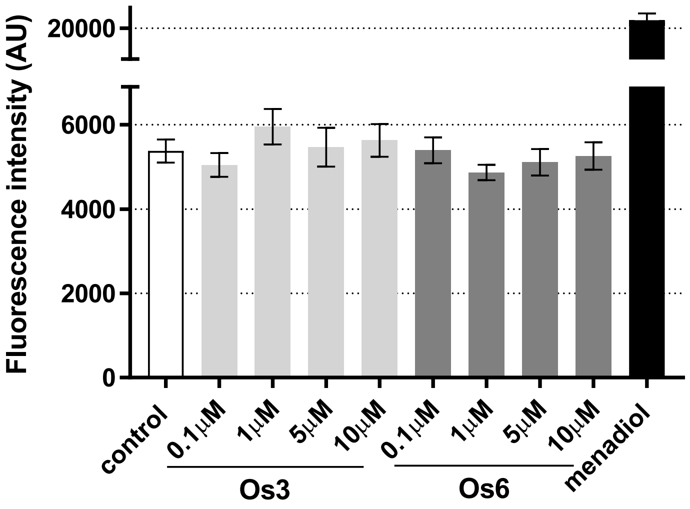

Figure S29. Intracellular ROS levels measured in MDA-MB-231 cells by CellROX® green assay. MDA-MB-231 cells were treated with increasing concentrations (0.1, 1, 5, and 10 μM) of Os complexes for 2 h in the dark. The generation of intracellular ROS was measured immediately after the treatment. The values shown in this figure represent the fluorescence intensity (median) in arbitrary units (AU).

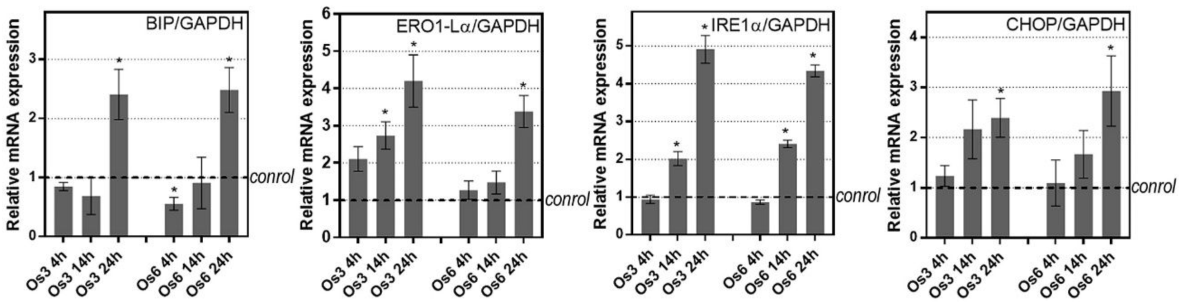

Figure S30. qRT-PCR of the ER stress markers in MDA-MB-231 cells treated with the investigated Os(II) complexes. Cells were treated for 4, 14, or 24 h at the concentration corresponding to $3xIC_{50,72h}$. Relative quantification of gene expression was calculated using the $2^{-\Delta\Delta Ct}$ method. The GAPDH gene was used as the endogenous reference control, and samples isolated from the untreated cells were used as the arbitrary calibrators. Data were subjected to statistical analysis by using a student's t-test, and the stars indicate a significant difference ($p \leq 0.01$) from untreated control samples (dashed line).

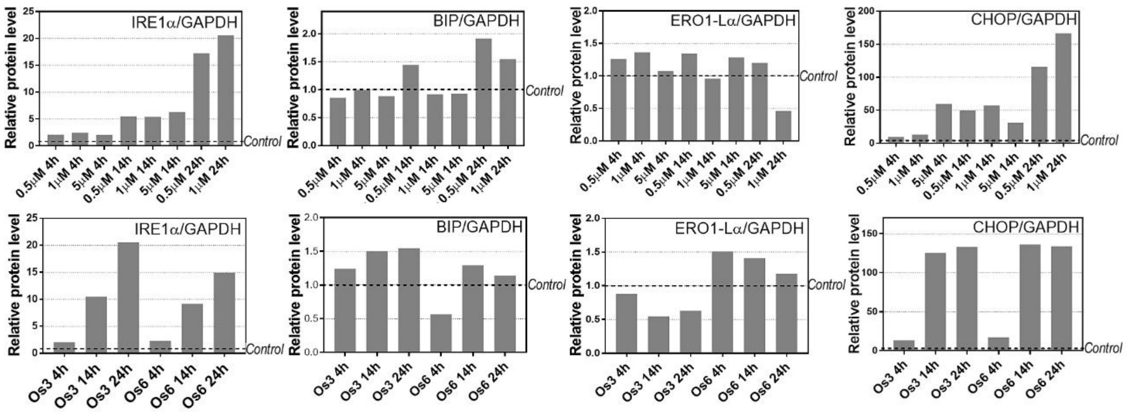

Figure S31. Quantitative evaluation of Western blotting data: the relative expression of ER stress markers normalized to GAPDH. Quantitative evaluation of Western blotting data: the relative expression of ER stress markers normalized to GAPDH.Top panels: MDA-MB-231 cells were treated with **Os3** complex for the indicated time and at indicated concentrations. Bottom panels: MDA-MB-231 cells were treated with **Os3** and **Os6** at their equitoxic concentrations corresponding to 3x$IC_{50,72h}$ for the indicated time. Protein levels are expressed relative to control, untreated cells (dashed line).
